# Transcription factor ASCL2 is required for development of the glycogen trophoblast cell lineage

**DOI:** 10.1371/journal.pgen.1007587

**Published:** 2018-08-10

**Authors:** Aaron B. Bogutz, Rosemary Oh-McGinnis, Karen J. Jacob, Rita Ho-Lau, Ting Gu, Marina Gertsenstein, Andras Nagy, Louis Lefebvre

**Affiliations:** 1 Department of Medical Genetics, Molecular Epigenetics Group, University of British Columbia, Vancouver, BC, Canada; 2 Lunenfeld-Tanenbaum Research Institute, Mount Sinai Hospital, Toronto, ON, Canada; Stanford University School of Medicine, UNITED STATES

## Abstract

The basic helix-loop-helix (bHLH) transcription factor ASCL2 plays essential roles in diploid multipotent trophoblast progenitors, intestinal stem cells, follicular T-helper cells, as well as during epidermal development and myogenesis. During early development, *Ascl2* expression is regulated by genomic imprinting and only the maternally inherited allele is transcriptionally active in trophoblast. The paternal allele-specific silencing of *Ascl2* requires expression of the long non-coding RNA *Kcnq1ot1* in *cis* and the deposition of repressive histone marks. Here we show that *Del*^*7AI*^, a 280-kb deletion allele neighboring *Ascl2*, interferes with this process in *cis* and leads to a partial loss of silencing at *Ascl2*. Genetic rescue experiments show that the low level of *Ascl2* expression from the paternal *Del*^*7AI*^ allele can rescue the embryonic lethality associated with maternally inherited *Ascl2* mutations, in a level-dependent manner. Despite their ability to support development to term, the rescued placentae have a pronounced phenotype characterized by severe hypoplasia of the junctional zone, expansion of the parietal trophoblast giant cell layer, and complete absence of invasive glycogen trophoblast cells. Transcriptome analysis of ectoplacental cones at E7.5 and differentiation assays of *Ascl2* mutant trophoblast stem cells show that ASCL2 is required for the emergence or early maintenance of glycogen trophoblast cells during development. Our work identifies a new *cis*-acting mutation interfering with *Kcnq1ot1* silencing function and establishes a novel critical developmental role for the transcription factor ASCL2.

## Author summary

By controlling precise networks of target genes, transcription factors play important roles in cell fate determination during development. The *Ascl2* gene codes for a transcription factor essential for the maintenance of progenitor cell populations able to differentiate into specialized cell types in the intestine and in the extra-embryonic trophoblast lineage. The trophoblast is an essential component of the placenta, an organ required for development of the embryo in placental mammals. *Ascl2* belongs to a group of unusual genes, called imprinted genes, which are expressed from only a single parental copy. *Ascl2* is only expressed from the maternally inherited copy in the trophoblast, the paternal copy being kept silent. Here, we describe an engineered deletion neighboring *Ascl2* that interferes with the complete silencing of the paternal copy of the gene. We show that the low amount of ASCL2 produced from this deletion can rescue the embryonic lethality associated with non-functional maternal copies of *Ascl2*. Although the rescued embryos can often survive to term, their placenta is highly disorganized and lacks members of a specific cell lineage, the trophoblast glycogen cells. By analyzing the transcriptional profile of mutant trophoblast progenitors *in vivo* and of differentiated trophoblast stem cells, we show that ASCL2 plays a very early role in the formation of this cell lineage.

## Introduction

The *Ascl2* gene—previously known as *Mash2*—was originally cloned as a mammalian homologue of *Drosophila achaete-scute* genes and codes for a group A bHLH transcription factor [1,2]. In mice, *Ascl2* is required for development. Homozygous *Ascl2* knock-out mice die at ~E10 due to an extra-embryonic phenotype which can be rescued by providing a functional tetraploid trophoblast lineage [3]. More recently, *Ascl2* was shown to play critical roles in a number of other cell types and developmental processes, including intestinal stem cells [4], epidermal development [5], myogenesis [6], and follicular T-helper cells [7]. ASCL2 acts by hetero-dimerization with ubiquitous bHLH factors of the E protein family, such as TCF3/E12-E47, TCF4/E2-2/ITF2, and TCF12/HEB/ALF1 [4,6,8]. These studies have shown that ASCL2-E-protein complexes promote transcriptional activation, although during myogenesis ASCL2 inhibits the action of bHLH myogenic factors by sequestration of E proteins [6], suggesting context-dependent mechanisms for ASCL2 function.

*Ascl2* is an imprinted gene in the mouse, expressed only from the maternal allele in trophoblast cells [9]. Consequently, embryos inheriting a maternal null allele (denoted *Ascl2*^*KO/+*^) fail to develop past mid-gestation, whereas paternal heterozygotes (*Ascl2*^*+/KO*^) are fully viable. Imprinting at *Ascl2* is tissue-specific: it is biallelically expressed in LGR5-positive intestinal stem cells [4], although the mechanism involved in this tissue-specific loss of imprinting (LOI) is still unknown. In trophoblast, the paternal allele of *Ascl2* is silenced by the paternally expressed long non-coding RNA (lncRNA) *Kcnq1ot1*, which bidirectionally establishes a repressive epigenetic domain in *cis*, covering more than 600 kb on distal Chr7 [10–15]. As a consequence, *Ascl2* is only expressed from the maternal allele, as are seven other protein-coding genes in the region [16].

*Ascl2* transcripts are deposited in the oocyte and zygotic transcription starts at the late two-cell stage [17]. Despite this early expression, *Ascl2* is first required only postimplantation, since maternal and zygotic mutants are both embryonic lethal at ~E10 [3,17]. Following implantation, the polar trophectoderm (TE) proliferates under FGF signaling from the underlaying epiblast [18]. These few cells will form a population of extra-embryonic ectoderm cells from which all the trophoblast cell types of the placenta will differentiate, with the exception of primary parietal trophoblast giant cells (P-TGCs), which are derived from the mural trophectoderm [19]. As it expands in the proximal-distal axis, this mass of diploid trophoblast cells forms two morphologically distinct structures: (*i*) the ectoplacental cone (EPC), surrounded by secondary P-TGCs, derived from precursors in the EPC by endoreduplication, that are in direct contact with the decidua basalis; and (*ii*) the extra-embryonic ectoderm (ExE), extending from the EPC down to the epiblast. From E6.5 to E7.5, high levels of *Ascl2* are mostly restricted to diploid cells of the EPC, as FGF4 and Nodal signaling from the epiblast prevents high levels of expression in the stem cell compartment of the ExE and its derivative, the chorionic ectoderm (ChE) [20–22]. With the occlusion of the ectoplacental cavity and the concomitant loss of stem cell potential in ChE between E8.0 and 8.5 [23], *Ascl2* remains highly expressed in the EPC but is also detected throughout most of the ChE [8,17]. Subsequently, from E9.0 to E12.5, *Ascl2* is detected in specific derivatives of the EPC and the ChE, namely spongiotrophoblast (SpT) cells of the junctional zone (Jz) and some labyrinth trophoblast of unknown cell type, respectively [8,17,24].

The phenotype of *Ascl2*-deficient conceptuses is characterized by an inability to maintain diploid precursors within the EPC and a consequent absence of *Tpbpa-*positive Jz cells at E9.5 [3]. In the absence of *Ascl2*, multipotent EPC cells appear to default to the P-TGC lineage, resulting in an expansion of the P-TGC layer. Consistent with a transient role in specific multipotent diploid EPC and SpT cells, *Ascl2* levels decline past E12.5 and no expression is detected in differentiated derivatives of those cells, such as glycogen trophoblast (GlyT) cells and three giant cell lineages: P-TGCs, canal TGCs (C-TGCs), and spiral artery-associated TGCs (SpA-TGCs) [25–27].

**Fig 1:**
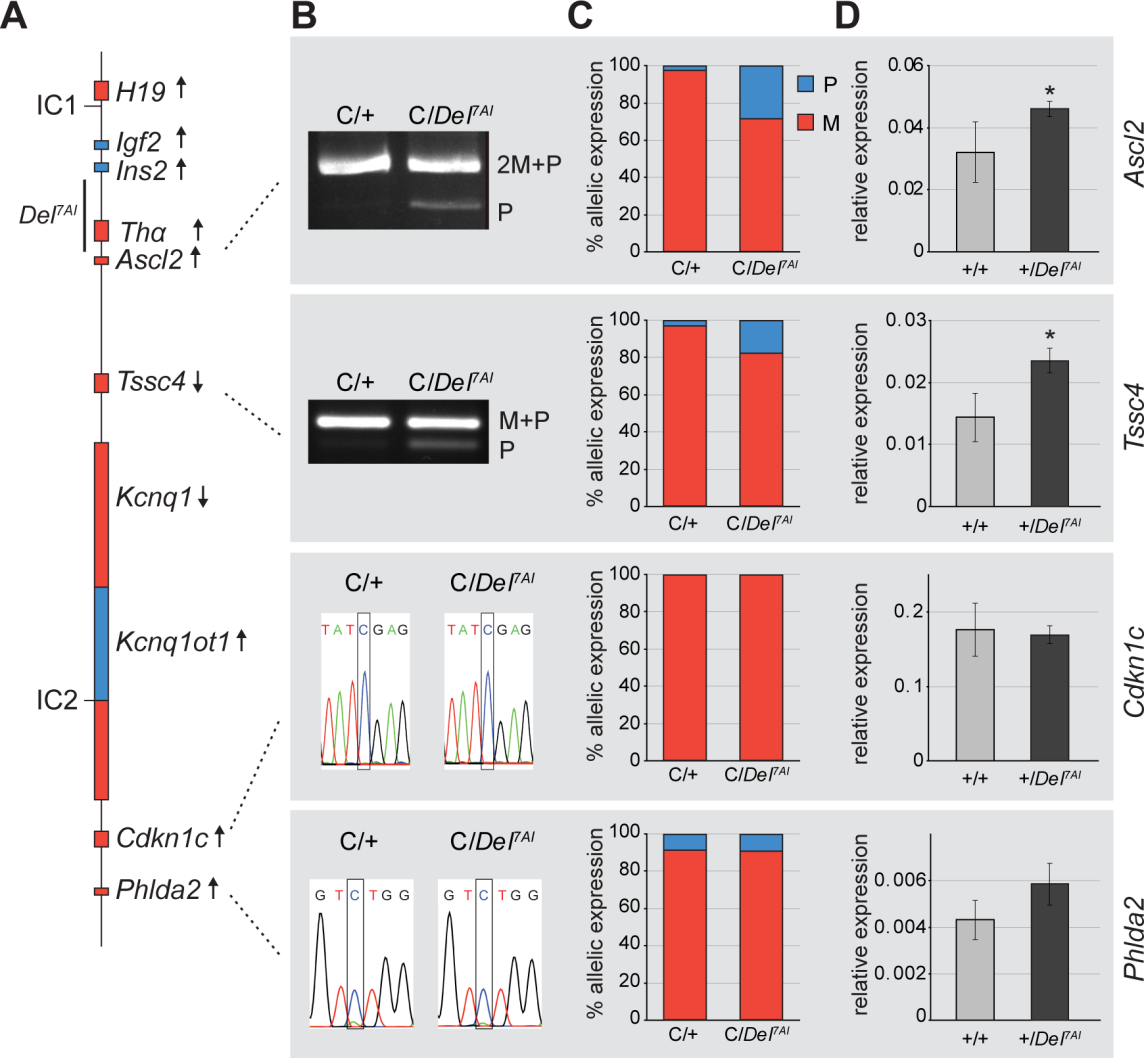
Loss of imprinting at *Ascl2* in +/*Del*^*7AI*^ placentae. (A) Simplified map of the imprinted region on distal mouse Chr7. Maternally expressed genes are shown in red, paternally expressed genes in blue. The gametic differentially methylated regions acting as imprinting centres (IC) in the *H19* and *Kcnq1ot1* sub-domains are labelled as IC1 and IC2, respectively. Location of the *Del*^*7AI*^ deletion is shown on the left, transcriptional orientation of each gene (arrows), on the right. (B) Allele-specific expression analysis for *Ascl2*, *Tssc4*, *Cdkn1c*, and *Phlda2*, on E13.5 placental cDNA. M and P show the position of the maternal and paternal bands, respectively. The 2M+P band contains two maternal bands and 1 paternal band; M+P, co-migrating maternal and paternal bands. Informative SNPs are boxed. For the *Cdkn1c* SNP, CAST = C and 129 = T; and for *Phlda2*, CAST = C and the 129 = A. C: WT CAST allele; +: WT 129 allele. (C) Maternal to paternal allele expression ratio (M to P ratio) for wild-type (C/+) and paternal deletion mutants (C/*Del*^*7AI*^). (D) Total expression levels in E15.5 placentae determined by RT-qPCR for three biological replicates per genotype. Error bars: SD for biological replicates. Expression is relative to *Ppia* levels. *p<0.005.

We previously described the generation of a ~280-kb deletion spanning the *Ascl2-Ins2* intergenic region and encompassing the gene for tyrosine hydroxylase, *Th* (Fig 1A) [28]. This deletion—called *Del*^*7AI*^—does not interfere with the establishment or maintenance of differential DNA methylation at the two neighbouring imprinting centers (ICs) regulating, amongst other genes, the lncRNAs *H19* and *Kcnq1ot1*. However, maternal transmission of *Del*^*7AI*^, recovered at the expected Mendelian ratio, leads to an intrauterine growth restriction phenotype [24]. *Del*^*7AI*^/+ placentae express *Ascl2* at ~60% of wild-type levels, are highly disorganized, but can still support development to term. As in the full *Ascl2* KO, these *Ascl2* hypomorphs show an expanded P-TGC layer but here a very thin Jz is maintained throughout gestation. Consequently, the labyrinth layer in these mutant placentae show an important proximal expansion of the fetal vasculature, suggesting a role for the Jz in restricting growth of the placental vessels [24]. In the mature placenta, the Jz is normally composed of two distinct diploid cell types, SpT and GlyT cells, both derived from the EPC [27]. Although *Del*^*7AI*^/+ placentae maintained a small layer of SpT cells, we could not detect GlyT cells in these mutants, suggesting a role for ASCL2 in the differentiation and/or maintenance of this trophoblast lineage [24]. The GlyT cell type was previously thought to be established past mid-gestation due to the emergence of vacuolated cells within the Jz at ~E12 that also express junctional zone-specific genes such as *Tpbpa* [29–31]. However, specific markers of these mature GlyT cells, notably *Pcdh12* and *Aldh1a3*, have since been shown to be expressed in a subset of EPC cells at E7.5 and E8.5, respectively, suggesting an earlier origin for this lineage [32,33].

Here we report that paternal inheritance of *Del*^*7AI*^ interferes with silencing at *Ascl2 in cis*, causing partial LOI and providing enough *Ascl2* mRNA to rescue maternally inherited *Ascl2* mutations. The rescued conceptuses share several phenotypic characteristics with our previously described *Del*^*7AI*^/+ hypomorphs, but show an even more pronounced placental phenotype, associated with lower rates of postnatal survival. Our analysis of these mutant placentae, together with RNA-seq profiling of E7.5 EPCs and differentiation assays of *Ascl2-*deficient trophoblast stem cells (TSCs), show that ASCL2 plays an essential role in the early specification or maintenance of GlyT cell precursors in the EPC during development, establishing a novel important function for this transcription factor in the trophoblast lineage.

## Results

### Partial loss of imprinting at *Ascl2* in paternal +/*Del*^*7AI*^ heterozygotes

Paternal transmission of *Del*^*7AI*^ is not associated with any obvious developmental or postnatal phenotypes [28]. However, expression studies suggested that total *Ascl2* levels might be increased in +/*Del*^*7AI*^ placentae at E9.5 compared to their wild-type littermates [24]. We first asked whether *Del*^*7AI*^ interferes with the epigenetic silencing of *Ascl2* and other *Kcnq1ot1*-regulated genes when paternally inherited (Fig 1A). For these allele-specific studies, we crossed +/*Del*^*7AI*^ males with females homozygous for a *Mus mus*. *castaneus* (C) distal Chr7 haplotype on the C57BL/6J background to obtain C/*Del*^*7AI*^ and C/+ embryos. We analyzed the ratios of expressed SNPs at four *Kcnq1ot1* targets—*Ascl2*, *Tssc4*, *Cdkn1c*, and *Phlda2—*from E13.5 placental RNA. Our results provide direct evidence of abnormal paternal *Ascl2* expression in C/*Del*^*7AI*^ placentae, indicating that *Ascl2* is not properly silenced from the paternal *Del*^*7AI*^ allele and exhibits partial loss of imprinting (LOI) (Fig 1B). We also noticed slight paternal expression for the neighboring gene *Tssc4*, although in wild-type conceptuses this gene is not tightly imprinted, as also shown by others [34]. In contrast, the more distal imprinted genes *Cdkn1c* and *Phlda2* showed no indication of LOI in the mutant placentae (Fig 1B). Maternal to paternal expression ratios confirmed that partial LOI was occurring at *Ascl2* and *Tssc4*, but not at *Cdkn1c* or *Phlda2* in paternal heterozygous *Del*^*7AI*^ placentae (Fig 1C). We also assessed total levels of gene expression by RT-qPCR and found corresponding significant increases in total *Ascl2* (1.4-fold) and *Tssc4* (1.6-fold) mRNA levels in E15.5 +/*Del*^*7AI*^ placentae compared to wild types, but not for the two other genes analyzed (Fig 1D). LOI at *Ascl2* was also quantified by blotting of restriction fragment length polymorphism analysis and confirmed using a strain-specific primer (S1 Fig). Together, our results show that the paternal allele of *Ascl2* is expressed at ~30% of the maternal allele levels in +/*Del*^*7AI*^ heterozygotes, representing ~23% of the total *Ascl2* levels in the mutants.

We previously showed that *Kcnq1ot1* is expressed and normally imprinted in +/*Del*^*7AI*^ embryos [28]. However, our analysis only documented transcription close (1.3 kb) to its transcription start site (TSS). The main stable and polyadenylated isoform of *Kcnq1ot1* spans ~83 kb and covers parts of introns 11 and 10 of *Kcnq1* [35]. However, transcripts extending all the way to ~121 kb and ~470 kb downstream of the TSS and requiring a functional *Kcnq1ot1* promoter have also been reported, although their abundance relative to the shorter isoform is conflicting [35,36]. Nevertheless, since the polyadenylated 470-kb isoform extends 130 kb within the region deleted in *Del*^*7AI*^ (S2A Fig), this raised the possibility that its production from the deletion allele might be perturbed in +/*Del*^*7AI*^ mutants showing partial LOI at *Ascl2*. Using primer pairs amplifying regions at 0.3, 202, and 307 kb from the *Kcnq1ot1* TSS, we were able to replicate the published data [36] and confirm transcription up to 21.3 kb upstream of *Ascl2* (307k PCR reaction) in wild-type E13.5 placenta (S2B Fig). Expression of the longer *Kcnq1ot1* isoform was also detected in +/*Del*^*7AI*^ placentae, arguing against a major interference with the production of the lncRNA up to the deletion breakpoint (S2B Fig).

Importantly, *Igf2* mRNA levels and expression pattern are not perturbed in the mutants, arguing against a spreading of *Kcnq1ot1* silencing effects to the paternally expressed *Igf2* gene in *cis*, across the deletion breakpoints (Fig 1A and S3 Fig). This result is consistent with the observation that +/*Del*^*7AI*^ mice do not exhibit an abnormal growth phenotype [28].

### Elevated *Ascl2* levels in differentiated +/*Del*^*7AI*^ trophoblast stem cells

To develop a cell culture-based system for further analyses of this deletion, we established trophoblast stem cell (TSC) lines from blastocysts carrying a maternally or paternally inherited *Del*^*7AI*^ allele. *Ascl2* levels peak between days 1 and 3 of TSC differentiation induced by FGF4 withdrawal [18,21,37–40]. Furthermore, RNA-seq profiling of hybrid TSC lines showed that *Ascl2* is already imprinted in undifferentiated TSCs, with more than 90% of expression coming from the maternal allele [41]. We used RT-qPCR to quantitate *Ascl2* mRNA levels in wild-type and *Del*^*7AI*^ heterozygous TSCs after 2 days of differentiation by culture in the absence of FGF4 and conditioned medium (S4A Fig). The paternal *Del*^*7AI*^ allele causes a significant increase in total *Ascl2* levels in these differentiated mutant cell lines compared to wild-type cells (~1.6-fold), in support of our observations *in vivo* (Fig 1D), and consistent with LOI at *Ascl2* in +/*Del*^*7AI*^ mutant TSCs.

### Rescue of *Ascl2* deficiency by paternal inheritance of the *Del*^*7AI*^ allele

**Fig 2:**
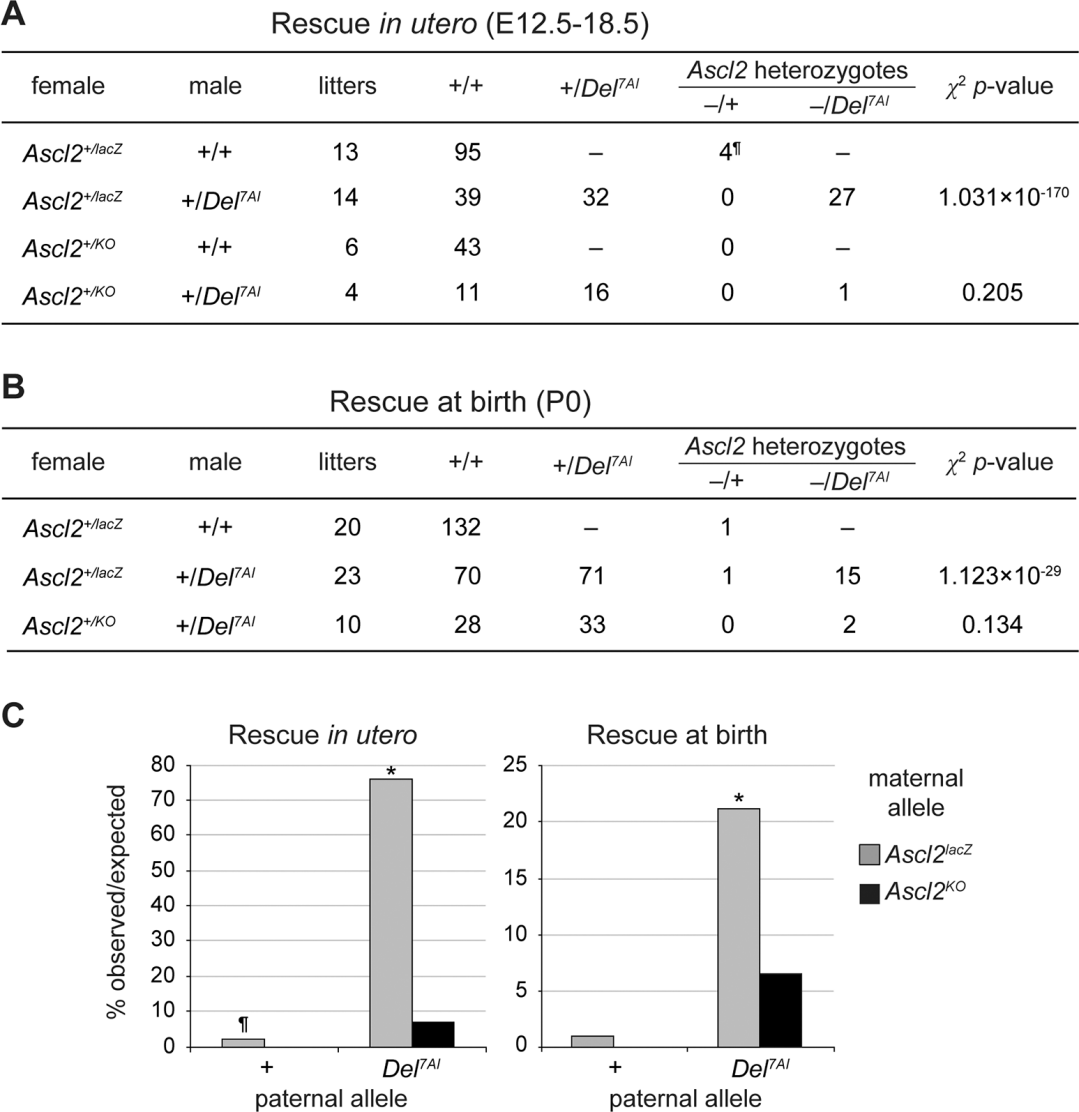
Phenotypic rescue of the mid-gestational lethality of *Ascl2* mutants by paternal inheritance of *Del*^*7AI*^. (A) Number of rescued embryos at E12.5 to E18.5 from crosses between *Ascl2*^*+/lacZ*^ or *Ascl2*^*+/*KO^ females and +/+ or +/*Del*^*7AI*^ males. ^¶^: includes 3 small and necrotic embryos. The data for individual litters are presented in S1 Table. (B) Number of live *Ascl2* mutant pups at birth obtained from similar crosses. (C) Bar charts showing the percentage observed over expected values from crosses analyzed *in utero* (left) and at birth (right). X-axis: male genotypes. Values from crosses involving *Ascl2*^*+/lacZ*^ and *Ascl2*^+/KO^ females are shown in grey and black, respectively. The probability values were obtained by the chi-square test of the null hypothesis of *Del*^*7AI*^ *=* WT, where the rescue frequency of the wild-type allele, 0.901%, is based on all crosses involving the *Ascl2*^*lacZ*^ allele (3/333 *Ascl2*^*lacZ/+*^ progeny). * *p*<1×10^−10^.

Maternal transmission of the *Ascl2* knock-in allele *Ascl2*^*lacZ*^ (official name *Ascl2*^*tm1*.*1Nagy*^) results in embryonic lethality at ~E10.0, as seen for the null allele [3], even though the bicistronic *Ascl2-*IRES-*lacZ* mRNA of this allele should produce a functional ASCL2 protein [42]. We found that the *Ascl2* mRNA from this allele is produced at only ~11% of wild-type levels in mutant *Ascl2*^*lacZ/+*^ ectoplacental cones at E7.5 (see below). We crossed *Ascl2*^*+/lacZ*^ females with +/*Del*^*7AI*^ males to determine if the small amount of paternal *Ascl2* expression from *Del*^*7AI*^ could rescue the embryonic lethality seen in *Ascl2*^*lacZ/+*^ mutants. We focused on *in utero* stages beyond the E10 lethality (E12.5–18.5) as well as on live pups the day following delivery (P0, Fig 2). Since *Ascl2*^*lacZ*^ is considered a weak, hypomorphic mutation [42], we also performed similar crosses with the original *Ascl2* null allele (*Ascl2*^KO^, official name *Ascl2*^*tm1Alj*^) to assess the effects of total *Ascl2* levels on our results [3]. First, we observed that both mutant *Ascl2* alleles give very few *Ascl2*^*-/+*^ live revertants when crossed to wild-type males, a phenotypic rescue that would require activation of the normally silent paternal allele of *Ascl2*. No live *Ascl2*^*KO/+*^ embryos or pups were obtained from 20 litters (0/91.5, observed/expected). For the *Ascl2*^*lacZ*^ allele, 4 *Ascl2*^*lacZ/+*^ embryos were recovered beyond E12.5 from 13 litters from wild-type males, but 3 of these were small and necrotic, suggesting incomplete resorption. Furthermore, two live *Ascl2*^*lacZ/+*^ pups were recovered from 43 litters implicating wild-type and +/*Del*^*7AI*^ males, leading to an overall epigenetic reversion rate of ~0.9% (3/333) for *Ascl2*^*lacZ/+*^ progeny (Fig 2).

Our results show that the paternal *Del*^*7AI*^ allele acts as a strong suppressor of the *Ascl2*^*lacZ*^ phenotype, with more than 75% of the expected *Ascl2*^*lacZ*^/*Del*^*7AI*^ compound heterozygotes surviving past E15 (Fig 2A and S1.1 Table). This phenotypic rescue is sensitive to the overall levels of *Ascl2* mRNA since we observed a marked decrease in the developmental rescue of the null allele *Ascl2*^*KO*^ by *Del*^*7AI*^, which plummets to ~7%. We also found that for live pups, just over 22% of the expected *Ascl2*^*lacZ*^/*Del*^*7AI*^ mice survived to term, down from ~75% rescue *in utero*, suggesting that although they can develop through late gestation, several of these conceptuses do not survive perinatally. Consistent with this possibility, *Ascl2*^*lacZ*^/*Del*^*7AI*^ rescued embryos appeared normal, although growth retarded (see below), as late as E17.5 in gestation (S1.1 Table). Nevertheless, the observed frequencies of phenotypic rescue of the *Ascl2*^*lacZ*^ allele, but not of *Ascl2*^*KO*^, by *Del*^*7AI*^ are highly significant, both *in utero* and at birth (Fig 2, and S1 Table).

**Fig 3:**
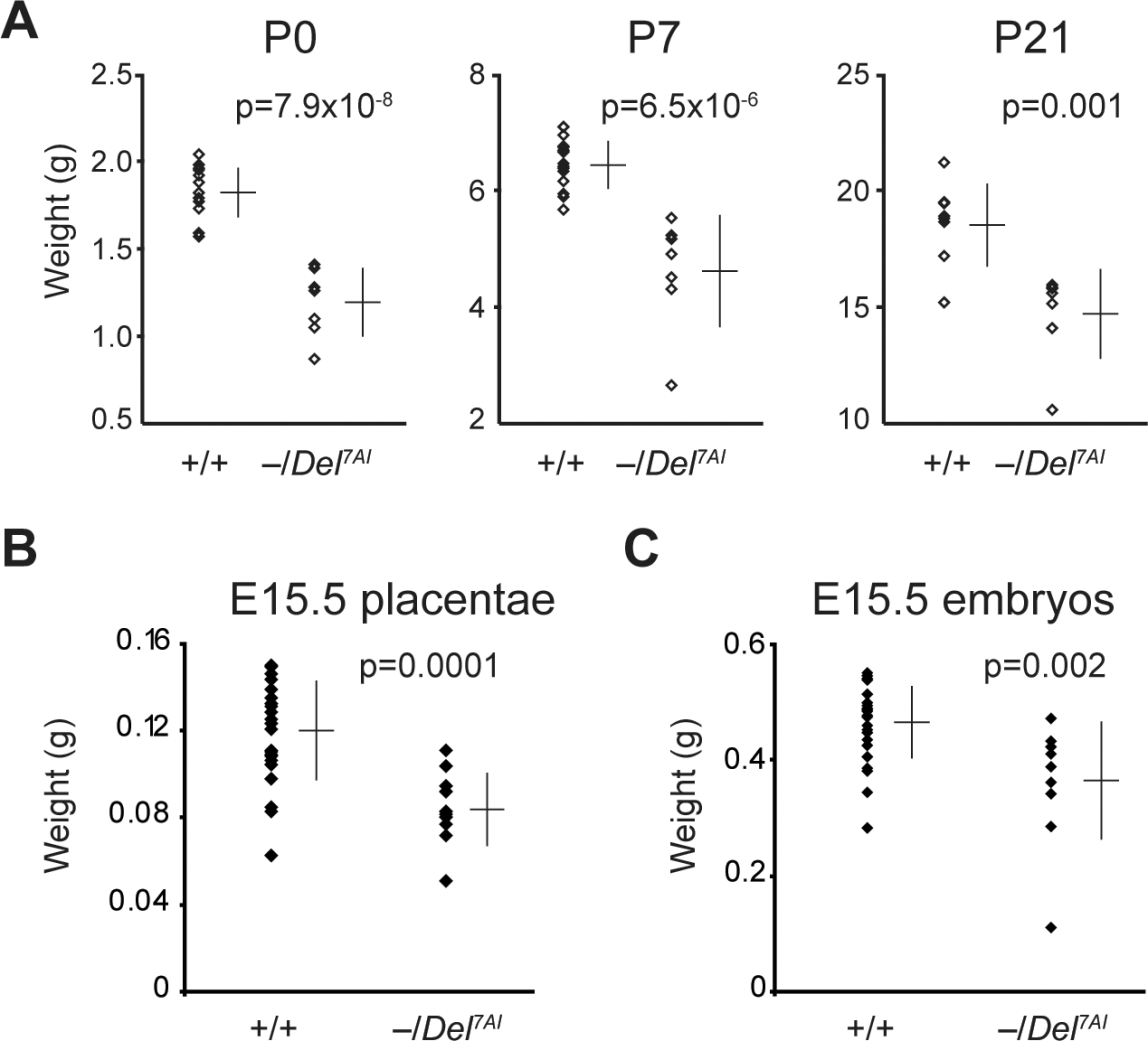
Growth retardation of rescued *Ascl2*^*lacZ*^/*Del*^*7AI*^ heterozygotes. (A) Scatterplots showing weights of rescued live *Ascl2*^*lacZ*^/*Del*^*7AI*^ pups (–/*Del*^*7AI*^, n = 7) and their wild-type littermates (n = 14) at postnatal days 0 (P0, birth), 7, and 21. Scatterplots of E15.5 placental (B) and embryonic (C) weights of *Ascl2*^*lacZ*^/*Del*^*7AI*^ conceptuses (–/*Del*^*7AI*^, n = 10) and wild type littermates (n = 25). In all graphs, the bars show the average weight ± SD.

Live rescued *Ascl2*^*lacZ*^/*Del*^*7AI*^ pups are growth-retarded and this growth phenotype largely persists until weaning age (Fig 3A). Since this growth retardation was already observed at birth, we measured the placental and embryonic weights of rescued *Ascl2*^*lacZ*^/*Del*^*7AI*^ conceptuses at E15.5. We noticed a significant (~29%) reduction in placental weight compared to their wild-type littermates (Fig 3B). Embryonic weights of the mutants were also smaller than wild-type littermates at this stage (~21%) (Fig 3C). We have previously reported that there is no difference in placental and embryonic weights in +/*Del*^*7AI*^ hemizygotes compared to wild-type littermates [24]. These rescue experiments provide genetic evidence that *Del*^*7AI*^ causes LOI at *Ascl2* and reveal a novel hypomorphic *Ascl2* phenotype in rescued, growth retarded conceptuses.

### *Ascl2*^*lacZ*^*/Del*^*7AI*^ placentae exhibit an expanded giant cell layer, a hypoplastic spongiotrophoblast, and lack glycogen trophoblast cells

**Fig 4:**
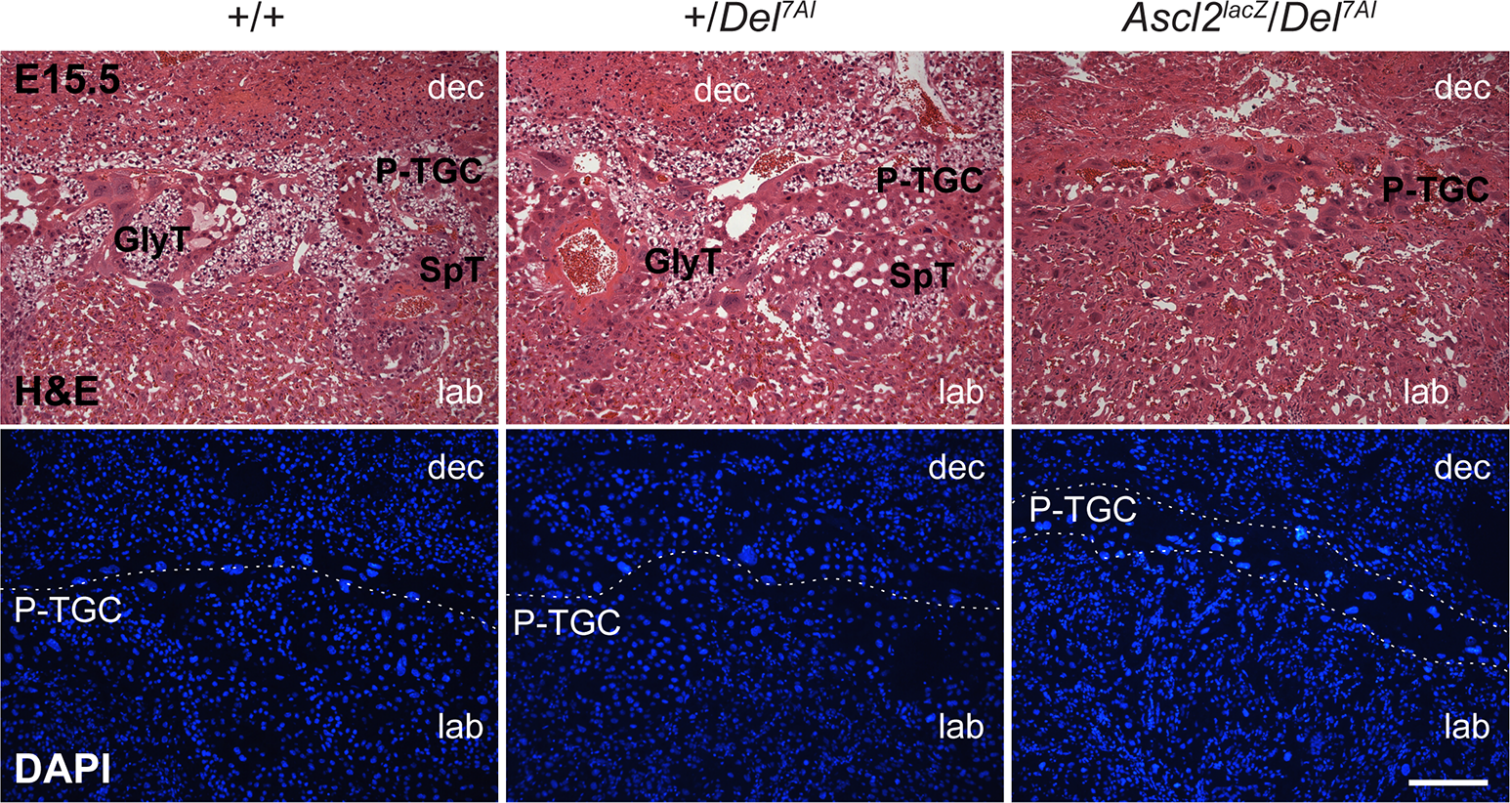
Hyperplastic parietal trophoblast giant cell layer in *Ascl2*^*lacZ*^/*Del*^*7AI*^ placentae at E15.5. Heamatoxylin and eosin (H&E, top) and DAPI (bottom) staining of wild-type, +/*Del*^*7AI*^ and rescued *Ascl2*^*lacZ*^/*Del*^*7AI*^ placental sections at E15.5. DAPI staining readily identifies the large polyploid nuclei of the cells stacked at the giant cell layer in the mutants. dec, decidua; P-TGC, parietal trophoblast giant cells; SpT, spongiotrophoblast cells; GlyT, glycogen trophoblast cells; lab, labyrinthine layer. Dashed lines indicate the P-TGC layer boundary. Scale bar: 0.5 mm.

The results presented above suggest that *Ascl2* levels are suboptimal and lead to a placental phenotype in rescued conceptuses. This possibility was first studied by histological analyses of placental sections at E15.5 (Fig 4). As described for the growth-retarded *Del*^*7AI*^/+ conceptuses [24], we observed an expansion of the parietal trophoblast giant cell (P-TGC) layer in rescued *Ascl2*^*lacZ*^/*Del*^*7AI*^ placentae. The histology data also suggest a reduction in number or absence of spongiotrophoblast and glycogen trophoblast (GlyT) cells in these mutants, the latter being present in both the Jz and decidua at this developmental stage in wild-type placentae [24,43]. These abnormalities were not observed in the paternal +/*Del*^*7AI*^ heterozygotes, which were indistinguishable from wild-type placentae (Fig 4).

**Fig 5:**
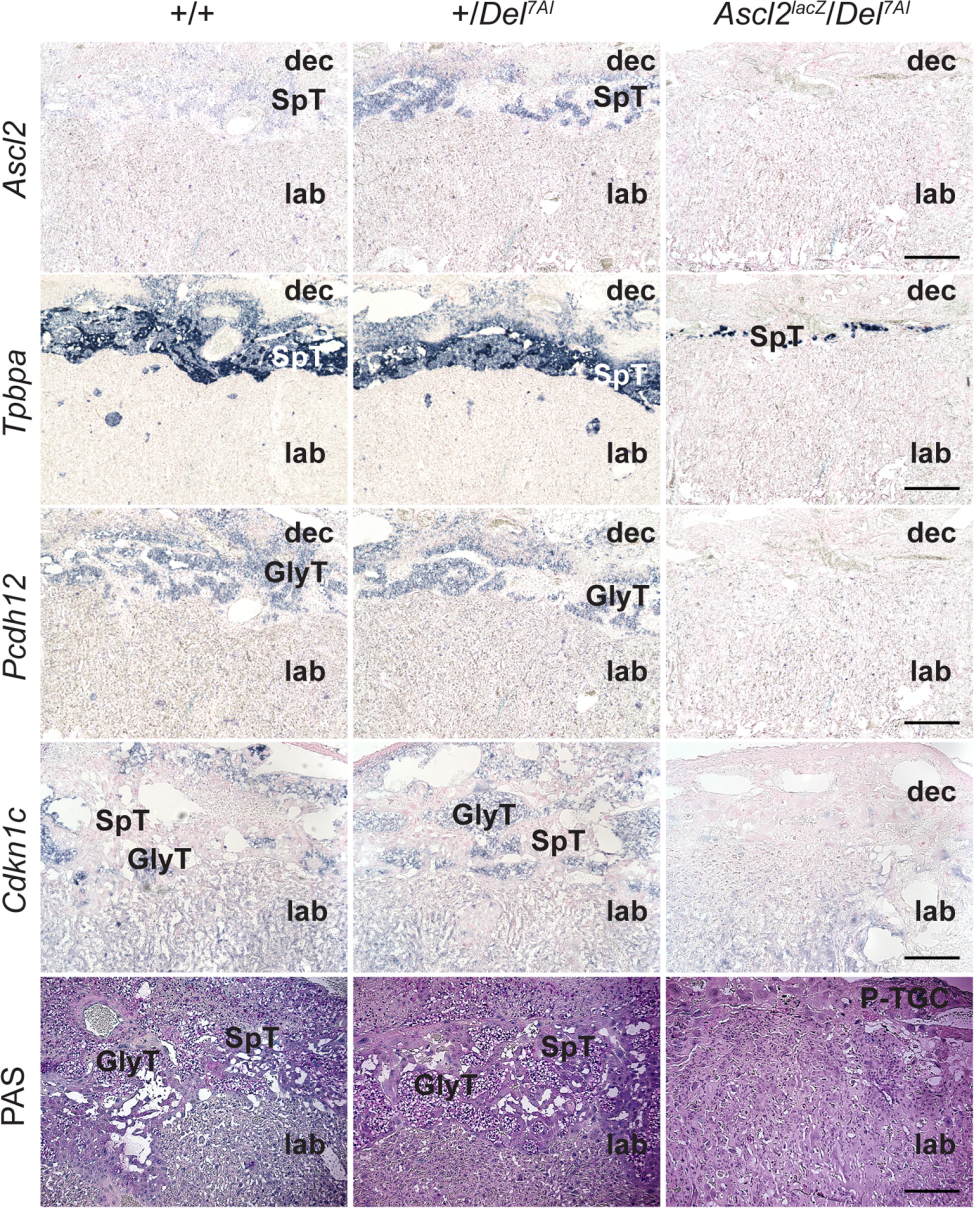
Abnormal spongiotrophoblast and glycogen trophoblast cell development in *Ascl2*^*lacZ*^/*Del*^*7AI*^ placentae at E15.5. Frozen sections of E15.5 placentae were analyzed for the expression of *Ascl2*, *Tpbpa*, *Pcdh12*, and *Cdkn1c* by *in situ* hybridization. Periodic acid-Schiff (PAS) staining of glycogen was performed on paraffin sections of E15.5 placentae. Multiple sections from two placentae of each genotype, labeled at the top, were assessed and representative pictures are shown. SpT, spongiotrophoblast cells; lab, labyrinthine layer; GlyT, glycogen trophoblast cells; P-TGC, parietal trophoblast giant cells; dec, maternal decidua. Scale bars: 0.5 mm.

**Fig 6:**
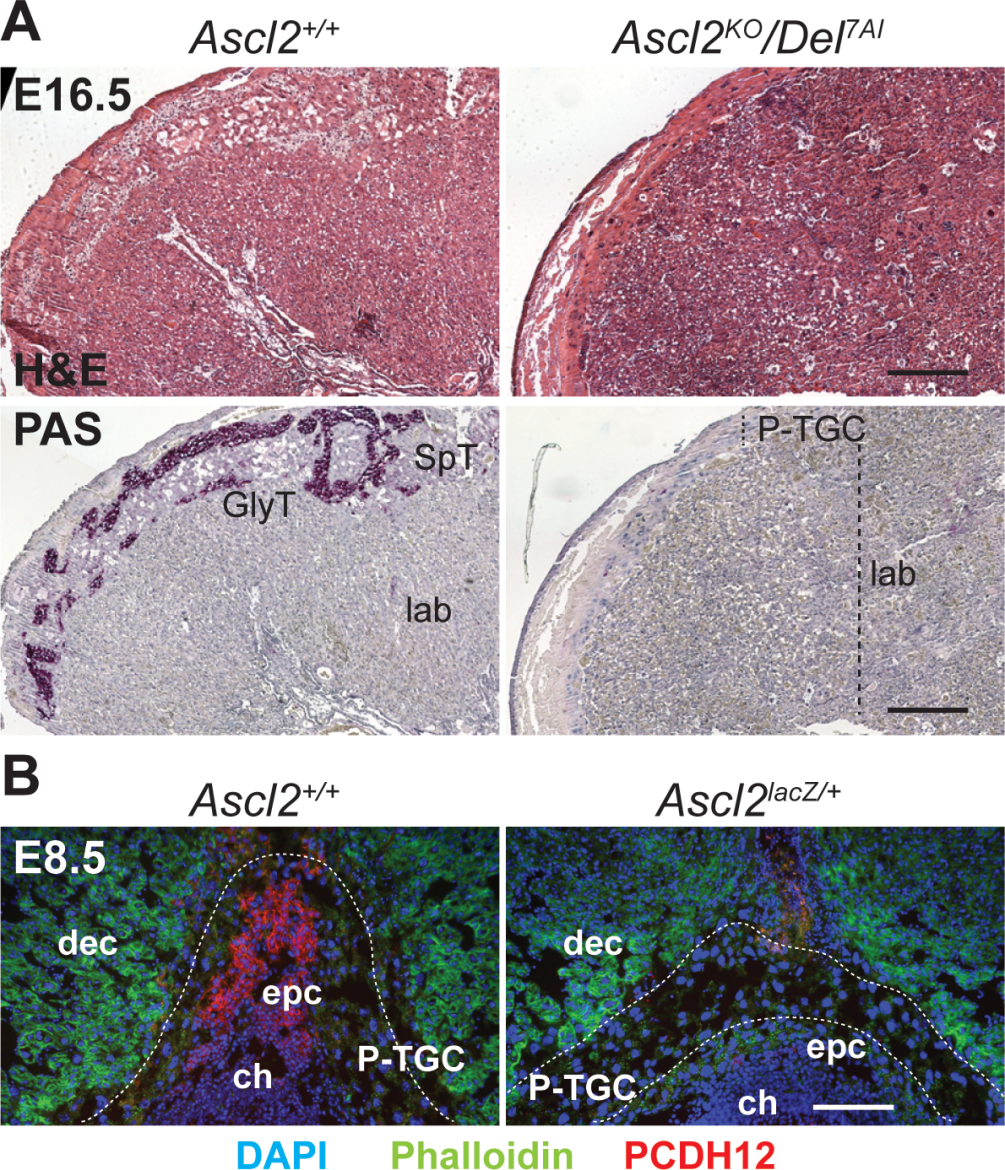
Absence of glycogen cell precursors in *Ascl2* mutant placentae. (A) Histological sections of wild-type and rescued *Ascl2*^*KO*^*/Del*^*7AI*^ placentae at E16.5 were stained with heamatoxylin and eosin (H&E, top) or PAS with heamatoxylin nuclear counter-stain (bottom). SpT, spongiotrophoblast cells; lab, labyrinthine layer; GlyT, glycogen trophoblast cells; P-TGC, parietal trophoblast giant cells. Scale bar: 0.5 mm. (B) Immunofluorescence staining with PCDH12 polyclonal antibodies (red) and phalloidin (green) on cryosections of E8.5 wild-type and *Ascl2*^*lacZ/+*^ conceptuses, counterstained with DAPI (blue). Scale bar: 0.1 mm.

To describe this phenotype in more detail we conducted a marker analysis by *in situ* hybridization on E15.5 placentae of wild-type, +/*Del*^*7AI*^, and rescued *Ascl2*^*lacZ*^/*Del*^*7AI*^ genotypes. First, our results confirmed higher levels of *Ascl2* expression in +/*Del*^*7AI*^ compared to wild-type spongiotrophoblast and further suggested a near-absence of *Ascl2-*expressing spongiotrophoblast cells in rescued placentae (Fig 5). Both of these observations are supported by RT-qPCR analyses (Fig 1D and S4B Fig). The near absence of a Jz was also confirmed by the severe reduction in *Tpbpa*-positive cells in rescued placentae (Fig 5). In addition to strong expression in SpT cells, *Tpbpa* is also expressed at lower levels in GlyT cells at this stage [30,31]. These cells represent an important fraction of *Tpbpa*-positive cells in wild-type and +/*Del*^*7AI*^ placentae at E15.5, readily seen in the decidua, but are absent in rescued *Ascl2*^*lacZ*^/*Del*^*7AI*^ conceptuses, which only show a small layer of SpT cells. Loss of signal for *Pcdh12* and *Cdkn1c* expression in these compound heterozygotes provided further support for the absence of GlyT cells (Fig 5). *Pcdh12* is exclusively expressed in GlyT cells in the placenta [32] whereas *Cdkn1c* labels both the labyrinth and GlyT cells at E15.5 [43,44]. Histological staining of glycogen content by periodic acid-Schiff (PAS) showed that GlyT cells are absent in E15.5 *Ascl2*^*lacZ*^/*Del*^*7AI*^ placentae (Fig 5) as well as in the single E16.5 *Ascl2*^*KO*^/*Del*^*7AI*^ rescued conceptus recovered (Fig 6A). At this later stage, the mutant placenta also shows little sign of SpT cells and an extended labyrinth layer reaching the hyperplastic P-TGC layer, with no sign of Jz. The presence of a dense and disorganized labyrinth reaching the P-TGC layer was also evident in the expression patterns of *Prl3b1*, *Gcm1*, and laminin (S5 Fig). Together our results show that as a consequence of reduced *Ascl2* levels the rescued mature placentae are severely hypoplastic for spongiotrophoblast cells and lack GlyT cells.

### ASCL2 is required for the emergence of glycogen trophoblast cells in the ectoplacental cone

Although the histological detection of glycogen has long been used to identify mature GlyT cells in the placenta past mid-gestation, few marker genes, such as *Pcdh12* and *Aldh1a3*, have been shown to label presumptive GlyT cell progenitors within the ectoplacental cone (EPC) at E7.5-E8.5 [32,33]. Since *Ascl2* is expressed at high levels in the EPC at those stages [3,42], we studied the requirement for ASCL2 in the emergence of this progenitor population. Using polyclonal anti-PCDH12 antibodies [45], the presumptive GlyT precursors are readily detected in the EPC of wild-type conceptuses at E8.5 (Fig 6B). As previously reported, we found that the EPC is already hypoplastic in *Ascl2*^*lacZ/+*^ mutants at this stage [3,42]. More importantly, the few remaining EPC cells seen in *Ascl2*^*lacZ/+*^ mutants are not positive for PCDH12 (Fig 6B), suggesting a failure to differentiate or maintain GlyT precursors in the absence of full *Ascl2* levels. Although PCDH12-positive cells were previously reported in the uterine crypt at E7.5 [32], we found that such staining, visible notably in the *Ascl2*^*lacZ/+*^ mutants, represent a primary antibody-independent background (S6 Fig).

**Fig 7:**
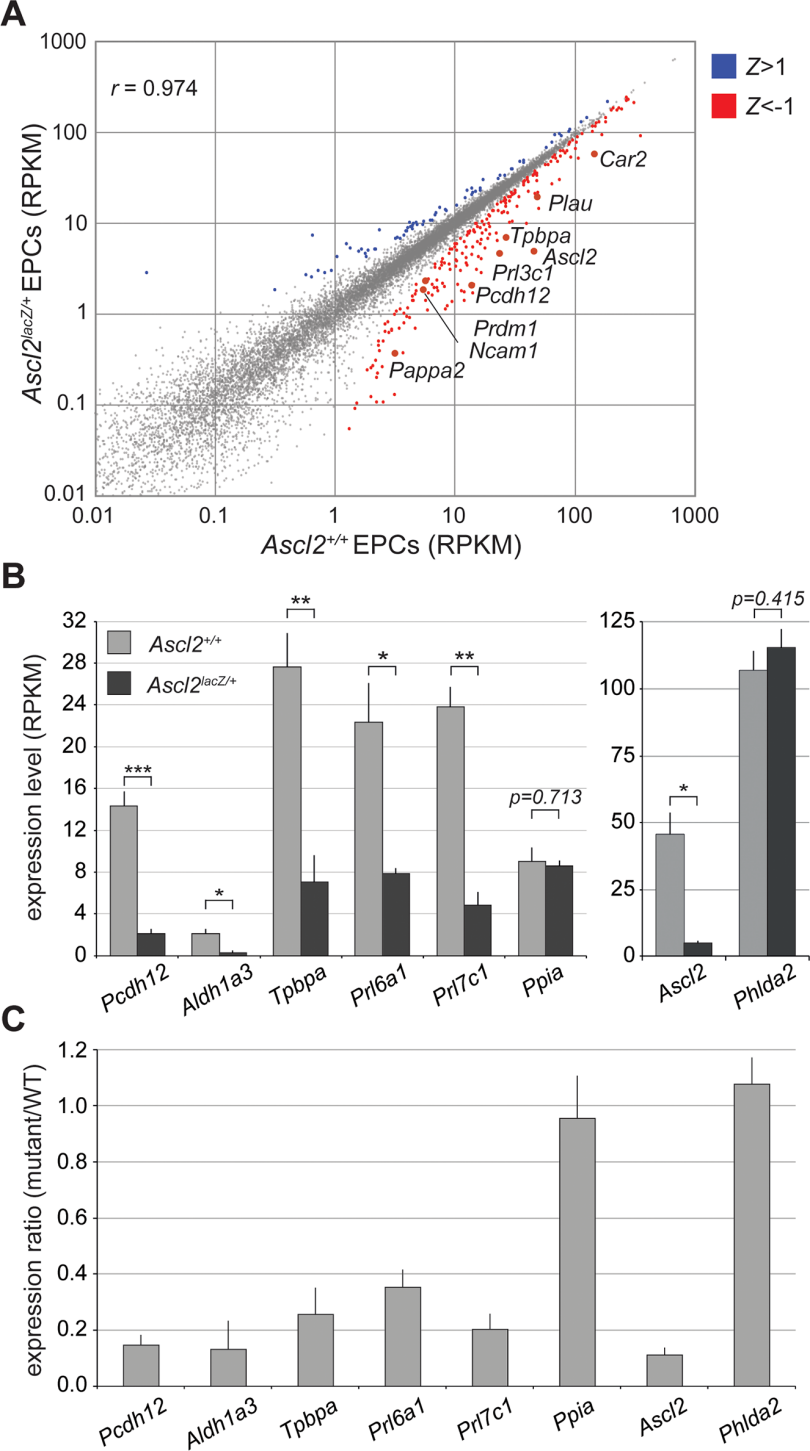
RNA-seq analysis of wild-type and *Ascl2-*deficient E7.5 ectoplacental cones. (A) Scatter plot showing average RPKM values for 25,071 autosomal RefSeq genes, from the RNA-seq analysis of dissected E7.5 EPCs from three wild-type (*Ascl2*^*+/*+^) and three mutant (*Ascl2*^*lacZ/*+^) littermates. Significantly upregulated genes (72 genes, *Z* > 1) and downregulated genes (217 genes, *Z* < −1) are shown in blue and red, respectively. *r*: Pearson correlation coefficient. (B) Average RPKM values for selected genes known to be expressed in spongiotrophoblast and glycogen trophoblast derivatives of EPC progenitors, from the RNA-seq analysis of E7.5 EPCs of given genotypes. *Ppia* is included as a housekeeping gene control. * *p*<0.05; ** *p*<0.01; *** *p*<0.005. (C) Fold change in RPKM values (mutant/wild-type) for the genes analyzed in B. Graphs show average and SD for three biological replicates per genotype.

To analyze global gene expression profiles prior to a morphologically visible phenotype, we mined RNA-seq datasets for wild-type and *Ascl2*^*lacZ/+*^ EPCs at E7.5 [46]. Overall, expression levels for most of the 25,071 autosomal RefSeq genes queried are highly correlated between the two genotypes (*r* = 0.9739, Fig 7A). We identified 217 significantly downregulated genes in the mutant EPCs (*Z*-score < −1) and 72 upregulated genes (*Z-*score > 1, Fig 7A and S2 Table). *Ascl2* itself is expressed at only ~11% of wild-type levels from the *Ascl2* hypomorphic allele in the mutant EPCs. Other significantly downregulated genes include markers of GlyT precursors in the EPC such as *Pcdh12* [32], *Aldh1a3* [33], and *Tpbpa* [31], as well as placental prolactin-related genes expressed in both E8.5 EPC and E14.5 GlyT cells, such as *Prl6a1*, and *Prl7c1* (Figs 7B and 7C, and S3 Table) [47]. Other genes significantly downregulated in the *Ascl2* mutant EPC and expressed in GlyT cells of the mature placenta include *Car2*, *Plau*, *Ncam1*, and *Prdm1/Blimp1* (Fig 7A) [48–50]. The RNA-seq data show that relatively few genes are affected at this early stage by the loss of more than 90% of *Ascl2* mRNA in the E7.5 EPC, but also that, significantly, several of the affected genes are known markers of the GlyT cell lineage.

### Markers of GlyT cell precursors are not induced upon differentiation of mutant *Ascl2*^*lacZ/+*^ TSCs

**Fig 8:**
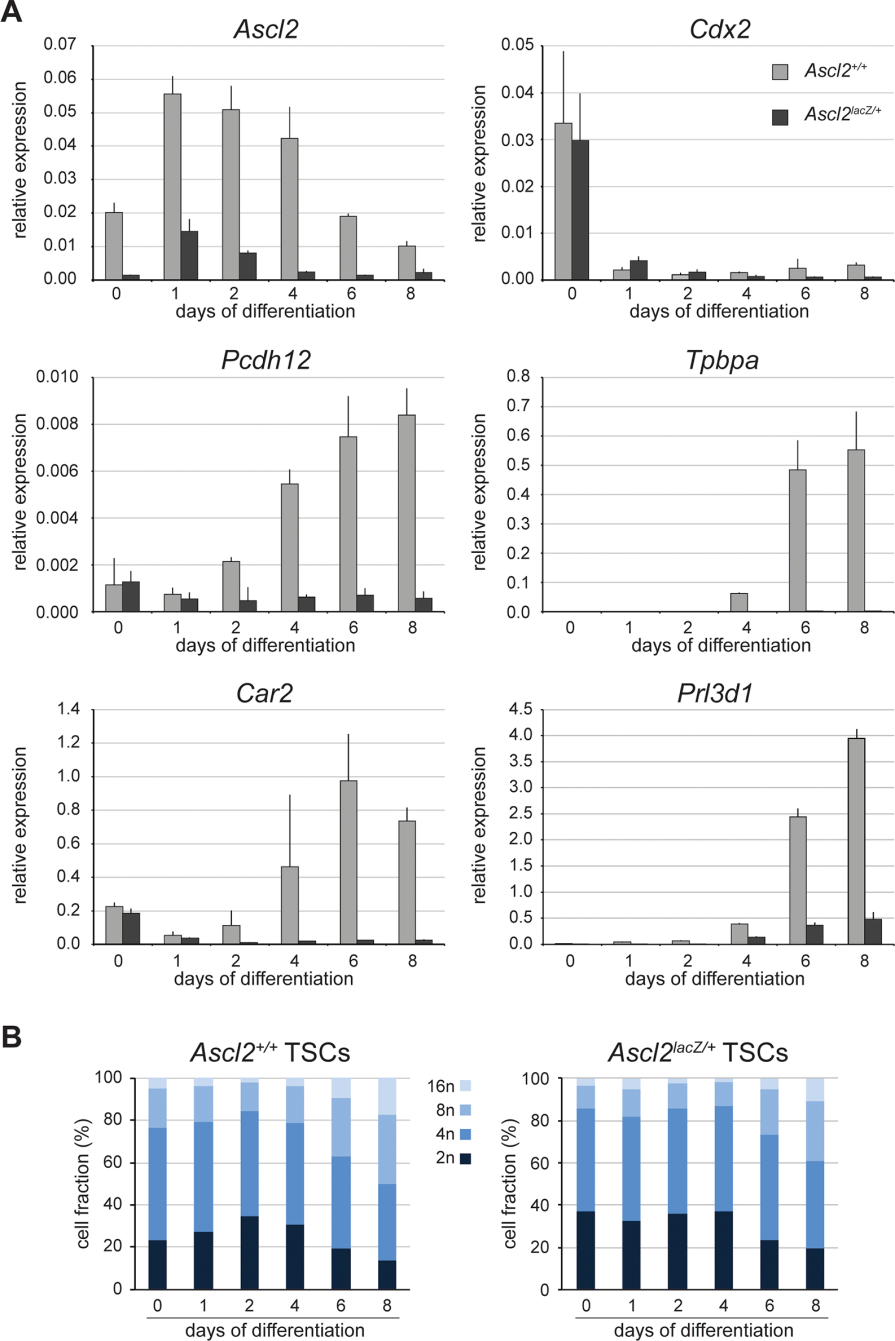
Differentiation defects of *Ascl2*^*lacZ/+*^ trophoblast stem cells. (A) Wild-type (*Ascl2*^***+/*+**^) and mutant (*Ascl2*^***lacZ/*+**^) TSCs were differentiated by FGF4 and conditioned medium withdrawal for 8 days and RNA samples were collected at the time points shown. Expression levels for *Ascl2*, *Cdx2*, *Pcdh12*, *Tpbpa*, *Car2*, and *Prl3d1* were analyzed by RT-qPCR and expressed as levels relative to those of the house-keeping gene *Ppia*. The graphs show the average ± SD for technical triplicates. (B) Flow cytometric analysis of ploidy during TSC differentiation, performed by DNA staining with propidium iodide. Cells of higher ploidy (8n and 16n) are seen in differentiated cells of both genotypes starting at day 6 of differentiation. FACS profiles are shown in S7 Fig.

The observation that *Ascl2*^*lacZ/+*^ EPCs lack PCDH12-positive cells *in vivo* and show broad defects in the expression of several GlyT cell markers suggest that ASCL2 might be required as early as E7.5 for the differentiation of this trophoblast lineage. To look at these early events, we established TSC lines from *Ascl2*^*+/+*^ and *Ascl2*^*lacZ/+*^ littermate blastocysts. Cell lines of both genotypes were recovered, with no visible differences in morphology or growth when maintained under undifferentiated growth conditions. *Ascl2* is expressed at ~14% of wild-type levels on average in the mutant TSC lines (Fig 8A), similar to the results obtained in EPCs (Fig 7B). This suggests that *Ascl2* is not required for the derivation or self-renewal of undifferentiated TSCs, consistent with the absence of morphological defects prior to E8.5 in *Ascl2-*null conceptuses [3]. Wild-type and mutant TSC lines were differentiated by FGF4 and fibroblast conditioned medium withdrawal for 8 days and lineage markers were analyzed by RT-qPCR. Within 24 hours of differentiation, lines of both genotypes showed a drastic decrease in the levels of *Cdx2* mRNA, a marker of undifferentiated TSCs (Fig 8A) [18]. Whereas *Pcdh12* [32], *Tpbpa* [30], and *Car2* [48] are induced within ~4 days of differentiation of wild-type TSCs, the mutant cells failed to activate these markers of GlyT (*Pcdh12*) and Jz cells (*Tpbpa*, *Car2*; Fig 8A). Although levels of the P-TGC marker *Prl3d1* [47] were reduced ~8-fold in the differentiated mutant TSCs, endoreduplication was not affected (Fig 8B and S7 Fig). The results show that *Ascl2* is required for the differentiation of GlyT precursors from undifferentiated TSCs, revealing a novel function for this bHLH transcription factor in early trophoblast development.

## Discussion

Glycogen trophoblast cells represent an abundant lineage of the mature mouse placenta although both the origin and function of these cells remain unknown. Originally characterized by their accumulation of large cytoplasmic stores of glycogen past mid-gestation, they were first detected as cell clusters within the Jz from ~E12.5 [29]. GlyT cells are migratory and starting at ~E14.5 they cross the P-TGC layer and invade the decidua, a property they share with SpA-TGCs [26,29]. Between E12.5 and E16.5 they are highly proliferative and expand more than 250-fold, while SpT cells increase by less than 4-fold [43]. More than 50% of these cells lose their glycogen content after E15.5. Consequently, it was proposed that GlyT cells might provide energy for embryonic growth late in gestation or for triggering parturition [29,43,51].

A number of observations suggest that GlyT precursor cells are already specified in the EPC. The most compelling evidence comes from the expression patterns of markers specific to GlyT cells in mature placentae and also expressed in specific cells of the EPC at E7.5 to 8.5, such as *Pcdh12* and *Aldh1a3* [32,33]. Several other markers are expressed in the EPC and later in GlyT cells of the mature placenta, but unlike *Pchd12* and *Aldh1a3*, these are also expressed in other trophoblast cell types. Cre/*lox*P-based cell lineage tracing experiments have been performed for two of those genes, *Tpbpa* and *Prdm1*, and confirmed their expression in GlyT amongst other trophoblast cell types in mature placentae [26,49]. However, Cre recombination and activation of the conditional reporters used were not documented in EPC, therefore leaving the possibility that GlyT cells were labelled only past mid-gestation, within the Jz. Another line of evidence comes from the direct detection of few glycogen-containing cells within the middle top portion of the EPC at E6.5–7.5 using histological PAS staining [32,52]. Together, these results suggest that the EPC contains different diploid populations, including precursors of GlyT cells initially found in the proximal portion of the EPC.

Here we show that the bHLH transcription factor ASCL2, which is broadly expressed in the EPC, is required for the presence of these GlyT cell precursors. Since heterodimerization with one of the E proteins is required for ASCL2 function [8], the subset of EPC cells developing as GlyT cell precursors may therefore be at least in part dictated by restricted expression of E protein genes. Both *Tcf4* and *Tcf12*, coding for the E proteins E2-2/ITF2 and HEB/ALF1, but not *Tcf3*, are expressed in the EPC at E8.5. However the available data suggest that both are mostly expressed in the distal portion of the EPC, closer to the chorion [8]. In contrast to those results, the RNA-seq data on individual wild-type EPC at E7.5 show that those three genes, including *Tcf3*, are expressed at this stage, with the following RPKM values: *Tcf3*, 6.74±0.31; *Tcf4*, 9.80±0.22; and *Tcf12*, 15.68±1.25 [46]. Mice deficient for any of these three E protein genes die before weaning, with no report of abnormalities suggesting a developmental phenotype, except perhaps for the *Tcf4*^*-/-*^ mice, which are recovered at low frequency at birth [53–55]. Whether the development of GlyT precursors is affected in any of those mutants, with the possibility of redundancy, still needs to be addressed.

Mature placentae developing from EPCs expressing *Ascl2* at suboptimal levels show a severe reduction in SpT cells and an absence of GlyT cells, as seen in *Del*^*7AI*^/+ placentae (~60% of wild-type *Ascl2* levels) [24] and in the current description of *Ascl2*^*lacZ*^*/Del*^*7AI*^ rescued placentae (~41% of wild-type levels). Our observations that several GlyT cell markers are downregulated in mutant EPCs before the manifestation of a visible phenotype and that *Ascl2-*deficient TSCs fail to upregulate *Pcdh12* and *Tpbpa* upon differentiation both support the conclusion that ASCL2 is required in diploid EPC cells for the emergence or early maintenance of the GlyT cell lineage. Together with previous observations [3,24,42] our results show that *Ascl2* function is level-sensitive in the trophoblast. Total *Ascl2* mRNA expression levels between ~60% and ~40% of wild-type levels are compatible with development to term but are associated with a severe placental phenotype leading to embryonic growth retardation. Although GlyT cells are deficient in these models and could contribute to the observed embryonic growth phenotype, we also described several other associated placental abnormalities which could contribute to an insufficient placenta. Specific ablation of the GlyT cell lineage using a conditional approach previously applied to other trophoblast lineages would be required to establish the developmental function of this cell population [56,57].

As *Ascl2* mRNA levels fall below ~60% of wild-type levels, the placenta fails to develop normally although it can still support the development of most growth restricted embryos to term (S8 Fig). Although the placentae of *Ascl2*^*lacZ*^*/Del*^*7AI*^ and *Del*^*7AI*^*/+* mutants shares similarities [24], the phenotype described here for *Ascl2*^*lacZ*^*/Del*^*7AI*^ mutants is more pronounced, with fewer persisting SpT cells, consistent with the lower overall *Ascl2* levels. The postnatal survival rate of *Ascl2*^*lacZ*^*/Del*^*7AI*^ mutant pups is consequently much lower (22% vs. 100%), although the cause of the late lethality is currently unknown [28]. At the molecular level, there are two additional distinctions between these two models; *i*) the maternally expressed form of placental tyrosine hydroxylase, *Th* [36,58], is deleted in *Del*^*7AI*^*/+* but not in *Ascl2*^*lacZ*^*/Del*^*7AI*^ mutants; *ii*) whereas *Phlda2* is upregulated in the *Del*^*7AI*^*/+* model [24], we found that *Phlda2* mRNA levels are similar in wild-type and rescued *Ascl2*^*lacZ*^*/Del*^*7AI*^ placentae at E13.5 (S4B Fig). The RNA-seq analysis of E7.5 EPCs also failed to detect a significant increase in *Phlda2* levels in the *Ascl2*^*lacZ/+*^ mutants, with RPKM values of 107.0±7.0 and 115.2±6.9 in the wild-types and mutants, respectively (*Z-*score = 0.551; Fig 7B and 7C). *Phlda2*, like *Ascl2*, is also maternally expressed and regulated by *Kcnq1ot1* [10,59]. Although overexpression of *Phlda2* (2.3- to 4-fold) from BAC transgenes causes a reduction in the volume of the junctional zone, as seen in *Ascl2* hypomorphs, the phenotype described here is much more pronounced and is also associated with an expansion of the P-TGC layer, not seen with increased *Phlda2* levels [60].

A recent study also described the consequences of overexpressing *Ascl2* by ~6-fold in the trophoblast via BAC transgenesis [61]. Although the authors reported a decrease in the average Jz volume at E14.5, when *Ascl2* levels are normally lower, the spongiotrophoblast and GlyT cell populations were not affected at earlier stages (E10.5–12.5). Importantly, the transgene leads to higher levels of *Ascl2* in the labyrinth, where it is normally expressed at low levels in a few scattered cells, and this overexpression is accompanied by ectopic clusters of PAS-positive, *Tpbpa*-positive cells within the labyrinth [61]. We propose that some of the expressing labyrinthine cells are able to acquire a GlyT cell phenotype at this ectopic location due to higher *Ascl2* levels. Those results thus provide additional evidence that *Ascl2* plays a critical role in the emergence of GlyT cells within the trophoblast. It would be important to precisely define the nature of the cell types expressing *Ascl2* in wild-type labyrinth and study its function in this cell population using a conditional mutagenesis approach [4].

Another important aspect of our work is the mechanism by which *Del*^*7AI*^ leads to partial LOI at *Ascl2*. Through our analyses *in vivo* and in mutant TSCs, we estimated that the paternal *Del*^*7AI*^ allele allows an abnormal expression of *Ascl2* representing ~30% of the wild-type mRNA levels transcribed from the maternal allele. This could imply that all +/*Del*^*7AI*^ ASCL2-positive cells express the paternal *Ascl2* allele at low levels (constitutive partial LOI) or that only ~30% of those cells experience full LOI and express *Ascl2* biallelically (mosaic full LOI). Our observations that +/*Del*^*7AI*^ placentae show broad overexpression of *Ascl2* in SpT cells and that much fewer than 30% of *Tpbpa-*positive SpT cells persist in *Ascl2*^*lacZ*^*/Del*^*7AI*^ rescued placentae (Fig 5) strongly support the former scenario. More importantly, unlike what is observed for the *Ascl2*^*lacZ*^ allele, the paternal *Del*^*7AI*^ allele essentially fails to rescue the KO allele (Fig 2). If the observed *Ascl2*^*lacZ*^*/Del*^*7AI*^ rescue was attributable to 30% of the cells expressing *Ascl2* at wild-type levels from the paternal *Del*^*7AI*^ allele (mosaic full LOI), we would expect similar rescue frequencies and phenotypes in both *Ascl2*^*lacZ*^*/Del*^*7AI*^ and *Ascl2*^*KO*^*/Del*^*7AI*^ conceptuses. Since our deletion can only rescue the *Ascl2*^*lacZ*^ allele, our results are consistent with the frequency of rescue being dependent on the overall levels of *Ascl2* mRNA per cell, with the paternal *Del*^*7AI*^ allele allowing *Ascl2* expression at ~30% of wild-type levels by constitutive partial LOI.

Silencing of the paternal allele of *Ascl2* is dependent on expression of the lncRNA *Kcnq1ot1* in *cis*, which is itself silenced on the maternal allele by a DNA methylation mark directly inherited from oocytes [10,13,62–64]. A conditional deletion of the *Kcnq1ot1* promoter showed that continuous expression of the lncRNA is required to maintain silencing of the paternal *Ascl2* allele *in vivo* [65]. The mechanism by which *Kcnq1ot1* silences *Ascl2* and other maternally expressed genes in the region is mediated in part by recruitment of histone modifying enzymes such as EHMT2/G9A, EZH2 (PRC2 component), and RNF2 (PRC1 component), and *cis* deposition of the repressive histone marks H3K9me2, H3K27me3, and H2AK119ub1 on the paternal allele of the repressed genes [11,14,15,66]. The most parsimonious explanation for the observed LOI at *Ascl2* is that the *Pgk-lox*P*-neo-*pA cassette present at the deletion junction, 4.3 kb downstream of *Ascl2*, interferes with some aspect of *Ascl2* silencing [28]. However, we have not detected any paternal *Ascl2* expression from the *Pgk-lox*P*-neo-*pA-*lox*P insertion allele (*Ascl2*^*M2*^) used to define the deletion breakpoint [28] when we analyzed *Ascl2*^*CAST/M2*^ placental RNA (S1A–S1C Fig). Furthermore, the *Ascl2*^*M2*^ allele could not rescue the embryonic lethality of the maternal *Ascl2*^*lacZ*^ mutation above the rate of reversion seen with a wild-type paternal allele (S1.3 Table).

Another possibility is that the *Del*^*7AI*^ deletion removes sequences required for spreading or stable maintenance of the *Kcnq1ot1* silencing domain all the way to *Ascl2*. A number of observations lend support to this hypothesis. First, RT-PCR evidence supported by RNA knock-down and promoter deletion suggests that the lncRNA *Kcnq1ot1* can extend up to 470 kb from its promoter, past *Ascl2* and *Th* (S2A and S9A Figs) [36]. Here, we have shown that the longer isoform of *Kcnq1ot1*, up to 307 kb, appears normally produced from the paternal *Del*^*7AI*^ allele, but obviously a large fraction of this isoform is missing because of the deletion (S9 Fig). Second, we and others showed that the *Th* gene, located in between *Ascl2* and *Ins2*, is expressed at low levels and only from the maternal allele in placenta and TSCs [36,58,67]. We found that placental *Th* is produced as a maternally-expressed chimeric transcript, initiating within an endogenous retroviral LTR element (RMER19A) located in between *Ascl2* and *Th*, and at least partially silenced via a *Kcnq1ot1-*dependent mechanism on the paternal allele [58]. Third, we have previously shown that a ubiquitous EGFP transgene inserted upstream of *Ins2*, at the site defining the proximal breakpoint of *Del*^*7AI*^, is maternally expressed in a *Kcnq1ot1*-dependent mechanism (S9B Fig) [58]. Fourth, an 800-kb YAC transgene of the *Kcnq1ot1* imprinted domain, extending from *Nap1l4* to past *Th* [68], can recapitulate appropriate imprinted expression of most genes present on the transgene when integrated at an ectopic genomic location [69]. Strikingly, unlike the other protein-coding imprinted genes on the construct, both *Ascl2* and *Tssc4* failed to be silenced upon paternal transmission of this YAC transgene. Since silencing of the same two genes is perturbed by *Del*^*7AI*^, and based on the observations described above, we propose that sequences in between *Th* and *Ins2* are required to establish a full functional silencing domain dependent on *Kcnq1ot1* transcription (S9D Fig). Further experimental support for such an extended domain of *Kcnq1ot1* silencing would require the analysis of *Kcnq1ot1*-dependent deposition of repressive histone marks in the region or the description of intra-chromosomal interactions between *Kcnq1ot1* lncRNA and sequences around the *Th* locus, as described for *Kcnq1* [66], and could lead to further understanding of the mechanism of silencing and spreading in this imprinted domain. Alternatively, 3’ end sequences missing from the longer *Kcnq1ot1* isoform expressed from *Del*^*7AI*^–and possibly from the YAC transgene as well–could be critical for establishment of a fully silenced domain on the paternal allele. *Kcnq1ot1* truncation alleles could potentially be exploited to delimit those putative critical elements of the lncRNA, although the available evidence suggests that transcription of this lncRNA can somehow bypass the termination signals of at least three genes: *Trpm5*, *Ascl2*, and *Th* (S9 Fig).

## Materials and methods

### Ethics statement

Mice were bred and maintained in the Centre for Disease Modelling, Life Sciences Institute, University of British Columbia, under pathogen-free conditions. All animal experiments were performed under certificates A07-0160, A11-0293, and A15-0181 from the UBC Animal Care Committee and complied with the national Canadian Council on Animal Care guidelines for the ethical care and use of experimental animals.

### Mice and genotyping

For all heterozygous genotypes, the maternal allele is always presented first. For simplicity, *Ascl2*^*–/+*^ *+/Del*^*7AI*^ compound heterozygotes are referred to as *Ascl2*^*–*^*/Del*^*7AI*^. The generation and genotyping of the *Del*^*7AI*^ (*Del*^*(7Ascl2-Ins2)1Lef*^; MGI ID:3662901) and *M2* (*Ascl2*^*tm2Nagy*^; MGI ID: 4399141) alleles were previously described [28]. The *Del*^*7AI*^ deletion spans a ~280 kb region, from a SpeI site 2.7 kb upstream of *Ins2* exon 1 to a NcoI site 4.3 kb downstream of *Ascl2* exon 3 (positions 142,682,271–142,962,499 on GRCm38/mm10) and deletes a single known protein-coding gene, *Th*. The junction contains a *Pgk-lox*P*-neo-pA* cassette transcribed on the (-) strand, like *Ascl2* and *Ins2* [28]. Two different *Ascl2* (*Mash2*) mutant alleles were used in this study. The original *Ascl2*^KO^ allele [3] deletes most of the *Ascl2* open reading frame and is therefore a null allele (*Ascl2*^*tm1Alj*^; MGI ID: 2153832). In the *lacZ* knock-in allele *Ascl2*^*lacZ*^ (*Ascl2*^*tm1*.*1Nagy*^; MGI ID:2155757) an IRES-*lacZ-*pA reporter inserted in the 3‘UTR of *Ascl2* destabilizes the bicistronic mRNA leading to embryonic lethality at E10.5, as seen in *Ascl2*^*KO/+*^ heterozygotes [42]. The animals in this study were all on the CD-1 outbred mouse background except for allele-specific studies, in which one parent is an incipient congenic for a *Mus mus*. *castaneus* (CAST/EiJ) distal chromosome 7 haplotype on a mixed CAST/EiJ × C57BL/6J background. Since they were originally derived from R1 ES cells [70], the mutant alleles are all on strain 129S1 or 129X1 Chr7 haplotypes. Weights of placentae and embryos were taken immediately upon dissection with as much of the liquid removed as possible before weighing.

### Allele-specific expression analysis and quantitative reverse transcriptase (RT-qPCR)

For allele-specific expression analyses of IC1- and IC2-regulated genes, random-primed cDNA was generated from E9.5 or E13.5 placental RNA as described previously [24]. For *Ascl2*, the SNP analyzed is a C to T transition within a HpaII site in the 3’ UTR (position 1471 of RefSeq mRNA NM_008554.3). RT-PCR with primers 1148F and 726R yielded a 725-bp fragment which upon HpaII digestion gives a CAST-specific 316-bp band and 129-specific 218- and 98-bp bands, in addition to common bands of 99 and 310 bp (S1 Fig). For *Tssc4*, RT-PCR with primers F1 and R35 followed by HaeIII digest give a CAST-specific 247-bp band and a 129-specific 159-bp band. For *Cdkn1c*, primers p57S and p574 give a 364-bp product and the CAST>129 SNP is C>T at position 316. For *Phlda2*, primers Ipl 1 and Ipl R2 yield a 578-bp product and the CAST>129 SNP is A>C at position 209. For *Cdkn1c* and *Phlda2*, direct sequencing of the PCR products followed by Phred analysis [71] was performed to determine allelic expression ratios. For total expression level determination, cDNAs were analyzed by RT-qPCR. Total RNA was purified using Trizol (Invitrogen) according to manufacturer’s directions and DNase-treated (RQ1, Promega) for at least 1 h to remove contaminating gDNA. RNA was reverse-transcribed with SSII (Invitrogen), using random primers (N_15_) according to the SSII protocol. Quantitative PCR was performed on a Step-One Plus Real time PCR system (Applied Biosystems) using Eva Green (Biotium). Ct values of three biological replicates, obtained by the LinReg PCR program [72], were averaged and used to calculate relative amounts of transcripts, normalized to levels of the housekeeping gene *Ppia* [73]. All primer sequences are available in S4 Table.

### In situ hybridization (ISH) and immunohistochemistry (IHC)

E13.5 and E15.5 placentae were dissected in PBS and fixed in fresh 4% paraformaldehyde/1×PBS (RNase-free) overnight at 4°C. For E9.5 placentae, entire conceptuses were fixed to ensure integrity of cryostat sections during the ISH procedure. Antisense and sense strand probes for *Ascl2*, *Tpbpa*/*4311*, *Pcdh12*, *Cdkn1c*, and *Igf2* were DIG-labeled and used for ISH on 10-μm cryostat sections from E13.5 and E15.5 placentae as previously described [25]. Nuclear fast red was used as the counterstain. The *Igf2* probe sequence was obtained from www.genepaint.org (Accession number NM_010514; probe 486) and amplified from E13.5 placenta cDNA. IHC for laminin was done as previously described [24]. Slides were treated to remove paraffin, hydrated, and blocked with 0.3% hydrogen peroxide for 30 minutes and then with 5% goat normal serum, 0.5% BSA in PBS-T. Slides were incubated with rabbit polyclonal anti-laminin (Sigma L9393, at a 1/50 dilution in serum blocking solution) overnight at 4°C. The next day, the secondary biotinylated goat anti-rabbit antibody (Jackson ImmunoResearch) was added at a dilution of 1/500 and incubated for 30 minutes. ABC (Vector) was added for 30 minutes and DAB for 1 minute. Sections were counterstained with hematoxylin and washed in Scott’s Tap water solution (2% MgSO_4_, 0.35% NaHCO_3_ in distilled water) to help sharpen the contrast. Sections were then dehydrated and mounted with Entellan mounting medium (EM Science) under glass coverslips.

### Periodic acid-Schiff (PAS) staining

Placental paraffin sections were treated to remove paraffin, hydrated, and oxidized in 0.5% periodic acid solution for 5 minutes. Slides were placed in Schiff Reagent for 15 minutes, counterstained with hematoxylin, and washed in Scott’s tap water solution to help sharpen the contrast. Sections were then dehydrated and mounted with Entellan mounting medium (EM Science) under glass coverslips. Consecutive sections of one placenta per genotype were examined.

### Immunofluorescence

For PCDH12 localization analyses, entire E8.5 conceptuses were processed for cryostat frozen sections as described [74]. The affinity-purified anti-PCDH12 polyclonal antibodies were prepared and used as described [45]. For F-actin staining, sections were washed with water, counterstained with phalloidin (1/400, Invitrogen) for 20 minutes at room temperature, washed again with water, and counterstained with 4’,6-diamindino-2-phenylindole (DAPI, 2 μg/ml, Sigma) for 5 minutes at room temperature. At early developmental stages, we found that staining for F-actin was much stronger in maternal cells, allowing a clear demarcation of the conceptus-uterus boundary. Sections were washed with water and mounted on glass slides under coverslips with Vectashield (Vector Labs). The samples were analyzed on a Leica DMI6000B inverted fluorescent microscope and images were captured and processed with a Q-imaging Retiga 4000R monochrome camera and Openlab (Improvision).

### Analysis of RNA-seq data

The NGS datasets for the EPC RNA-seq analysis [46] were downloaded from GEO (accessions GSM1613552-7) and aligned to the mouse genome (mm9) using STAR two-pass mapping for *de novo* splice-junction generation with default parameters [75]. Data were input into SeqMonk (http://www.bioinformatics.babraham.ac.uk/projects/seqmonk/) for further analysis, removing reads with a mapping quality score of less than 5. RPKM values were generated using SeqMonk’s RNA-Seq Quantitation Pipeline, normalizing read count by gene length.

### Trophoblast stem cells

All the TSC lines used in this study were established from E4.0 blastocysts, expanded, and maintained as described [18]. TSCs were grown in RPMI 1640 (Invitrogen) supplemented with 50 μg/ml penicillin/streptomycin, 20% fetal bovine serum (Wisent), 1 mM sodium pyruvate, 100 μM β-mercaptoethanol, 2 mM L-glutamine, 70% 3-day feeder-conditioned medium, 25 ng/ml human recombinant FGF4 (Sigma F2278) and 1 μg/ml heparin (Sigma H3149). Cells were collected for genotyping and sexing by PCR; only XY male cells were selected for expression studies. Differentiation of TSCs was induced by omission of FGF4 and fibroblast-conditioned medium in the culture. The flow cytometric analysis of ploidy during TSC differentiation was performed by DNA staining with propidium iodide as described [76]. All data were obtained on a BD LSRII running BD FACS DIVA and analyzed in FlowJo 9.5.

### Statistical analysis

For RT-qPCR, weight comparisons, and RNA-seq expression data, statistical significance was determined using the Student’s *t*-test (unpaired, two tailed distribution), and data were presented as mean ± SD. For the rescue experiments, the probability values were calculated using the chi-square test of statistical significance against the null hypothesis of *Del*^*7AI*^(or *M2*) = WT allele in its ability to rescue the embryonic lethality of maternal *Ascl2* mutations. The rescue frequency of the wild-type allele, 0.901%, is based on all crosses involving the *Ascl2*^*lacZ*^ allele (3/333 *Ascl2*^*lacZ/+*^ progeny) and was used to calculate the expected numbers of *Ascl2*^*–*^*/Del*^*7AI*^ or *Ascl2*^*–*^*/M2* progeny (S1.3 Table).

## Supporting information

S1 Fig Allele-specific expression of *Ascl2* in wild-type and *+/Del*^*7AI*^ placentae. (**A**) Diagram of the *Ascl2* 725-bp exons 2–3 RT-PCR product (primers 1 + 4) showing the positions of HpaII sites for the 129 and CAST (C) alleles with sizes of each fragment given in base pairs. The polymorphic HpaII site within the 3’ UTR is marked by an asterisk. (**B**) Non-radioactive blot of the HpaII-digested RT-PCR products, hybridized with a DIG-labelled probe from the 218-bp 129 HpaII band (shown in A). The RNA samples analysed are from E9.5 placentae of the given genotypes, where C is the maternal CAST allele and *M2*, the targeted PGK-*lox*P-neopA-*lox*P insertion (*Ascl2*^*tm2Nagy*^) used to define the distal breakpoint of the *Del*^*7AI*^ deletion. M, maternal; P, paternal. (**C**) Allelic ratios (maternal/paternal) for each sample, as determined by ImageJ analysis of the data presented in B. (**D**) Diagram of the *Ascl2* genome from exon 2 to 3, showing the positions of PCR primers for genomic (**E**) and RT-PCR (**F**) analyses. The asterisk marks the position of the polymorphic HpaII site. The reverse primer 3 (129R) is 129-specific at its 3’ terminal nucleotide. (**E**) Intron 2 to exon 3 PCR on genomic DNA from pure 129 and CAST mice as well as a CAST/Del7AI embryo (C/Δ). Lanes–and M are water controls and a 100-bp marker. (**F**) Exon 2 to exon 3, 129-specific RT-PCR on cDNA from wild type (C/+) and mutant (C/Δ) placentae. Lanes–, + and M are water control, a 129 *Ascl2* cDNA clone, and a 100-bp marker, respectively. PCR primers: 1, 1148F; 2, in2F1; 3, 129R (129-specific); 4, 726R. PCR primers used are shown at the bottom of each gel figure. Their sequences are given in S4 Table.
(PDF)

S2 Fig Expression of *Kcnq1ot1* in +/*Del*^*7AI*^ placentae. (**A**) UCSC Genome Browser screenshot for the *Kcnq1ot1* imprinted domain. From the top, the tracks show: (*i*) The positions of PCR reactions used by Golding (2011) to define the longest *Kcnq1ot1* isoform. (*ii*) The three PCR reactions used in this study. (*iii*) The extent of the *Del*^*7AI*^ deletion. (*iv*) The longer *Kcnq1ot1* isoforms reported by Golding (2011, ~470 kb)) and Redrup (2009, ~121 kb), as well as the more stable and annotated transcript of ~83 kb. All are transcribed on the (-) strand, from a transcriptional start site (TSS) within intron 11 of *Kcnq1*. Note that ~130 kilobases of the longest isoform are deleted in *Del*^*7AI*^. (*v*) UCSC genes annotated in the region, including *Ascl2*, located immediately distal of the *Del*^*7AI*^ breakpoint. (**B**) RT-PCR detection of *Kcnq1ot1* at 0.3, 202, and 307 kb downstream of the TSS, on E13.5 placental RNA from two +/*Del*^*7AI*^ and one wild-type control conceptuses. PCRs were performed on total RNA samples, with (+) or without (-) reverse transcriptase (RT) priming of cDNA with random primers (N_15_). C-: water control. C+: genomic DNA. The molecular weight ladder is the exACTGene 100bp ladder (Fisher Scientific).
(PDF)

S3 Fig Paternal *Igf2* expression is unaffected in +/*Del*^*7AI*^ placentae at E13.5. (**A**) *Igf2* RT-qPCR on wild type and +/*Del*^*7AI*^ E13.5 placental cDNA. Expression is relative to *Ppia*. Three biological replicates for each genotype were analysed. Graphs show mean ± SD.
(**B**) *Igf2* ISH on frozen sections of wild type and +/*Del*^*7AI*^ E13.5 placentae. Multiple sections from two placentae of each genotype were assessed and representative pictures are shown. The sense probe gave no signal (not shown). The blue stain shows *Igf2* expression, mostly in the junctional zone and GlyT cells in the decidua. Scale bar: 0.5 mm. jz, junctional zone; lab: labyrinth; dec, decidua.
(PDF)

S4 Fig Effect of *Del*^*7AI*^ on *Ascl2* mRNA levels in differentiated TSCs and rescued placentae. (**A**) Trophoblast stem cell (TSC) lines of the given genotypes were differentiated for 2 days by FGF4 withdrawal and *Ascl2* levels, normalized to *Ppia* levels, were measured by RT-qPCR. In paternal deletion mutants (+/*Del*^*7AI*^), total *Ascl2* levels are increased by 1.6-fold over wild-type TSCs (*, p<0.05). Graphs show mean + SD. The numbers of independent TSC lines of each genotype analysed (biological replicates) are given at the bottom (n =). (**B**) Relative levels of *Ascl2* and *Phlda2* in E13.5 wild-type and *Ascl2*^*lacZ*^*/Del*^*7AI*^ rescued placentae, determined as described in A. Three samples of each genotype were analysed and graphs show mean ± SD of biological triplicates (**, p = 0.0003).
(PDF)

S5 Fig Abnormal labyrinth development in *Ascl2*^*lacZ*^*/Del*^*7AI*^ placentae at E15.5. Frozen sections of E15.5 placentae of the given genotypes were analysed for the expression of *Prl3b1* and *Gcm1* by ISH. The basement membrane marker laminin was detected by IHC on paraffin sections. Scale bar: 0.5 mm. Spt, spongiotrophoblast cells; dec, decidua; P-TGC, parietal trophoblast giant cells; lab, labyrinthine layer.
(PDF)

S6 Fig Primary antibody-independent staining in the decidua. Adjacent sections of the *Ascl2*^*lacZ/+*^ E8.5 conceptuses analysed in Fig 6B were treated as described in this figure but without incubation with the anti-PCDH12 primary antibodies. Punctate staining for the secondary antibody (arrow) is still visible above the giant cell layer, within the decidua. P-TGC, parietal trophoblast giant cells; dec, decidua; ch, chorion.
(PDF)

S7 Fig Endoreduplication of differentiating wild-type and *Ascl2*^*lacZ/+*^ TSCs. (**A**) Cell-cycle distribution of wild-type and *Ascl2*^*lacZ/+*^ mutant TSCs as monitored by flow cytometry using propidium iodide staining. Profiles were generated on the indicated days following FGF4 and conditioned medium withdrawal. 2n marks diploid cells in G1 phase, whereas 4n represents a mixture of G2-phase diploid and G1-phase tetraploid cells. Endoreduplication is clearly seen at higher ploidies. (**B**) Images of wild-type and *Ascl2*^*lacZ/+*^ mutant TSCs at d0 and d4 of differentiation. Scale bar, 100 μm. Refers to data presented in Fig 8B.
(PDF)

S8 Fig Dosage-sensitive effects of *Ascl2* mRNA levels on placental phenotype. For each genotype, the approximate total *Ascl2* mRNA levels are presented as a percentage of the wild-type levels, set at 100%.
(PDF)

S9 Fig Potential critical region for extended *Kcnq1ot1* silencing. (**A**) Structure of the IC1-IC2 imprinted domains on distal mouse Chr7, drawn to scale. Paternally expressed genes are in blue, maternally expressed genes in red. Two isoforms of *Kcnq1ot1* have been described; the more stable form terminates within intron 10 of *Kcnq1*, whereas a longer form has been detected, extending all the way past *Th*, a gene maternally expressed in placenta from the LTR RMER19A (Jones, 2011). (**B**) The Tel7KI allele carrying a pCAGGS-EGFP reporter inserted upstream of *Ins2*. The EGFP is imprinted and maternally expressed in the embryo in a *Kcnq1ot1*-dependent manner (Jones, 2011). (**C**) *Del*^*7AI*^ allele, showing partial LOI at *Ascl2* and *Tssc4* upon paternal transmission. (**D**) YAC transgene showing appropriate imprinting of the IC2 domain, except at *Ascl2* and *Tssc4* (Cerrato, 2005). In both C and D, the 3’ end structure of the longer *Kcnq1ot1* isoform is unknown (question marks).
(PDF)

S1 Table S1.1 Table. Litters collected to assess the rescue of the *Ascl2*^*lacZ*^ mutation *in utero*.
S1.2 Table. Litters collected to assess the rescue of the *Ascl2*^*KO*^ mutation *in utero*.
S1.3 Table. Chi-square tests of rescue data.
(XLSX)

S2 Table S2.1 Table. Significantly down-regulated autosomal genes (Z-score < -1) identified by RNA-seq analysis of wild-type and *Ascl2*^*lacZ/+*^ mutant EPC at E7.5.
S2.2 Table. Significantly up-regulated autosomal genes (Z-score > 1) identified by RNA-seq analysis of wild-type and *Ascl2*^*lacZ/+*^ mutant EPC at E7.5.
(XLSX)

S3 Table RPKM values for all expressed *Prl* genes, from RNA-seq analysis of wild-type and *Ascl2*^*lacZ/+*^ mutant EPC at E7.5, as well as their reported expression pattern in the placenta. (XLSX)

S4 Table Primers used in this study. (PDF)
