# Supplementary material for: Transcription factor ASCL2 is required for development of the glycogen trophoblast cell lineage
Source: PLoS Genet. 2018 Aug 10;14(8):e1007587. doi: 10.1371/journal.pgen.1007587 (PMC6105033; doi:10.1371/journal.pgen.1007587)
Supplement: S1 Fig — (A) Diagram of the Ascl2 725-bp exons 2–3 RT-PCR product (primers 1 + 4) showing the positions of HpaII sites for the 129 and CAST (C) alleles with sizes of each fragment given in base pairs. The polymorphic HpaII site within the 3’ UTR is marked by an asterisk. (B) Non-radioactive blot of the HpaII-digested RT-PCR products, hybridized with a DIG-labelled probe from the 218-bp 129 HpaII band (shown in A). The RNA samples analysed are from E9.5 placentae of the given genotypes, where C is the maternal CAST allele and M2, the targeted PGK-loxP-neopA-loxP insertion (Ascl2tm2Nagy) used to define the distal breakpoint of the Del7AI deletion. M, maternal; P, paternal. (C) Allelic ratios (maternal/paternal) for each sample, as determined by ImageJ analysis of the data presented in B. (D) Diagram of the Ascl2 genome from exon 2 to 3, showing the positions of PCR primers for genomic (E) and RT-PCR (F) analyses. The asterisk marks the position of the polymorphic HpaII site. The reverse primer 3 (129R) is 129-specific at its 3’ terminal nucleotide. (E) Intron 2 to exon 3 PCR on genomic DNA from pure 129 and CAST mice as well as a CAST/Del7AI embryo (C/Δ). Lanes–and M are water controls and a 100-bp marker. (F) Exon 2 to exon 3, 129-specific RT-PCR on cDNA from wild type (C/+) and mutant (C/Δ) placentae. Lanes–, + and M are water control, a 129 Ascl2 cDNA clone, and a 100-bp marker, respectively. PCR primers: 1, 1148F; 2, in2F1; 3, 129R (129-specific); 4, 726R. PCR primers used are shown at the bottom of each gel figure. Their sequences are given in S4 Table. (PDF) [file pgen.1007587.s001.pdf]

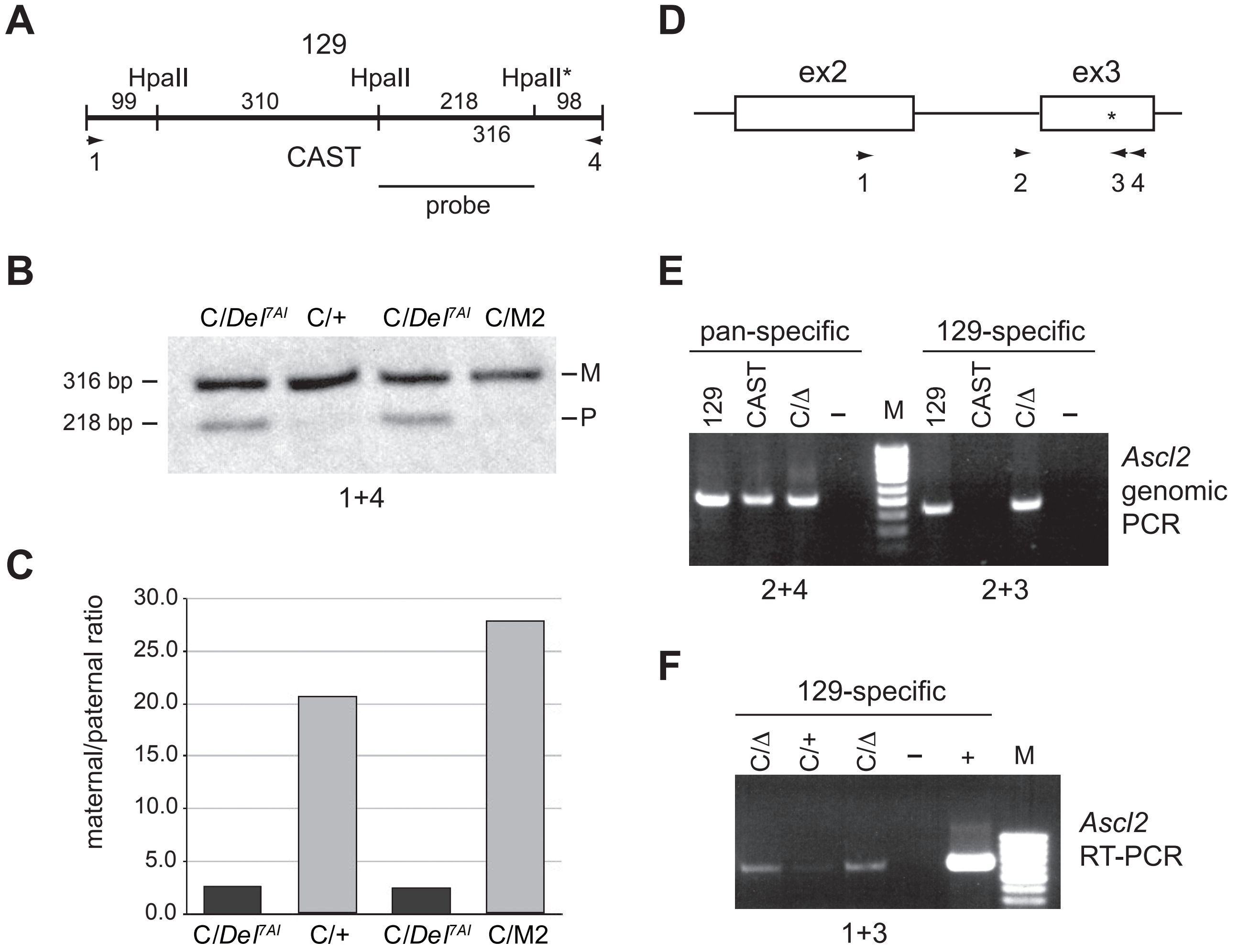

S1 Fig. Allele-specific expression of *Ascl2* in wild-type and +/*Del*^{7AI} placentae.
(A) Diagram of the *Ascl2* 725-bp exons 2-3 RT-PCR product (primers 1 + 4) showing the positions of HpaII sites for the 129 and CAST (C) alleles with sizes of each fragment given in base pairs. The polymorphic HpaII site within the 3' UTR is marked by an asterisk. **(B)** Non-radioactive blot of the HpaII-digested RT-PCR products, hybridized with a DIG-labelled probe from the 218-bp 129 HpaII band (shown in **A**). The RNA samples analysed are from E9.5 placentae of the given genotypes, where C is the maternal CAST allele and M2, the targeted PGK-*lox*P-neopA-*lox*P insertion (*Ascl2*^{tm2Nagy}) used to define the distal breakpoint of the *Del*^{7AI} deletion. M, maternal; P, paternal. **(C)** Allelic ratios (maternal/paternal) for each sample, as determined by ImageJ analysis of the data presented in **B**.
(D) Diagram of the *Ascl2* genome from exon 2 to 3, showing the positions of PCR primers for genomic (**E**) and RT-PCR (**F**) analyses. The asterisk marks the position of the polymorphic HpaII site. The reverse primer 3 (129R) is 129-specific at its 3' terminal nucleotide. **(E)** Intron 2 to exon 3 PCR on genomic DNA from pure 129 and CAST mice as well as a CAST/*Del*^{7AI} embryo (C/Δ). Lanes – and M are water controls and a 100-bp marker. **(F)** Exon 2 to exon 3 129-specific RT-PCR on cDNA from wild type (C/+) and mutant (C/Δ) placentae. Lanes –, + and M are water control, a 129 *Ascl2* cDNA clone, and a 100-bp marker, respectively. PCR primers: 1, 1148F; 2, in2F1; 3, 129R (129-specific); 4, 726R. PCR primers used are shown at the bottom of each gel figure. Their sequences are given in S4 Table.
