# Supplementary material for: Transcription factor ASCL2 is required for development of the glycogen trophoblast cell lineage
Source: PLoS Genet. 2018 Aug 10;14(8):e1007587. doi: 10.1371/journal.pgen.1007587 (PMC6105033; doi:10.1371/journal.pgen.1007587)
Supplement: S2 Fig — (A) UCSC Genome Browser screenshot for the Kcnq1ot1 imprinted domain. From the top, the tracks show: (i) The positions of PCR reactions used by Golding (2011) to define the longest Kcnq1ot1 isoform. (ii) The three PCR reactions used in this study. (iii) The extent of the Del7AI deletion. (iv) The longer Kcnq1ot1 isoforms reported by Golding (2011, ~470 kb)) and Redrup (2009, ~121 kb), as well as the more stable and annotated transcript of ~83 kb. All are transcribed on the (-) strand, from a transcriptional start site (TSS) within intron 11 of Kcnq1. Note that ~130 kilobases of the longest isoform are deleted in Del7AI. (v) UCSC genes annotated in the region, including Ascl2, located immediately distal of the Del7AI breakpoint. (B) RT-PCR detection of Kcnq1ot1 at 0.3, 202, and 307 kb downstream of the TSS, on E13.5 placental RNA from two +/Del7AI and one wild-type control conceptuses. PCRs were performed on total RNA samples, with (+) or without (-) reverse transcriptase (RT) priming of cDNA with random primers (N15). C-: water control. C+: genomic DNA. The molecular weight ladder is the exACTGene 100bp ladder (Fisher Scientific). (PDF) [file pgen.1007587.s002.pdf]

A

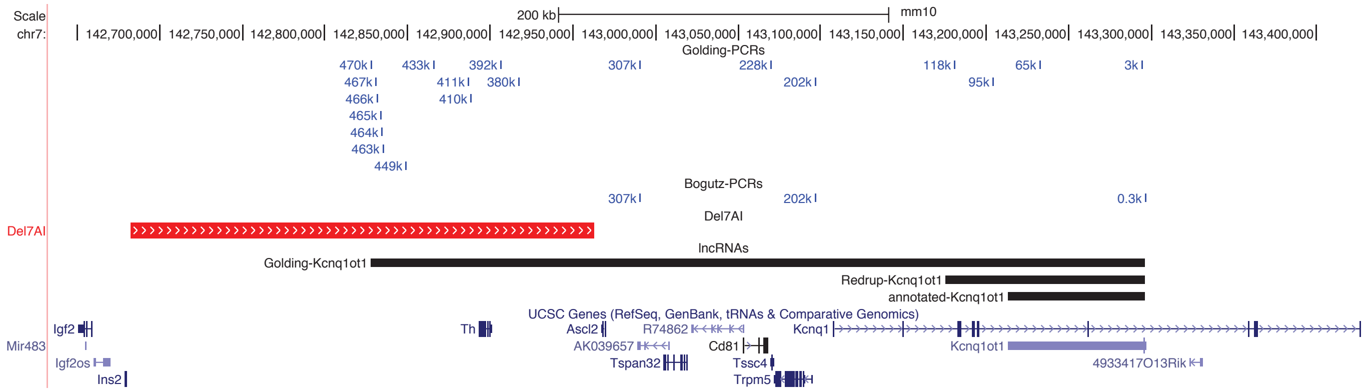

B

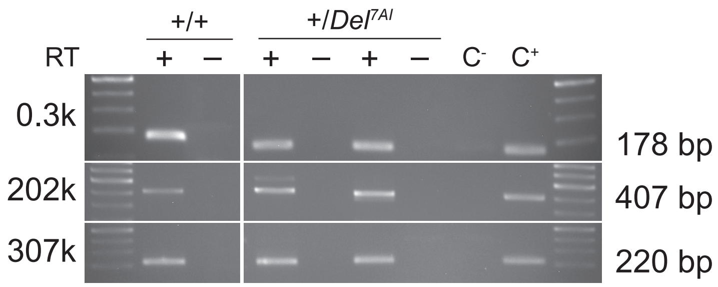

S2 Fig. Expression of *Kcnq1ot1* in +/*Del*^{7AI} placentae.

(**A**) UCSC Genome Browser screenshot for the *Kcnq1ot1* imprinted domain. From the top, the tracks show:

(*i*) The positions of PCR reactions used by Golding (2011) to define the longest *Kcnq1ot1* isoform.

(*ii*) The three PCR reactions used in this study.

(*iii*) The extent of the *Del*^{7AI} deletion.

(*iv*) The longer *Kcnq1ot1* isoforms reported by Golding (2011, ~470 kb)) and Redrup (2009, ~121 kb), as well as the more stable and annotated transcript of ~83 kb. All are transcribed on the (-) strand, from a transcriptional start site (TSS) within intron 11 of *Kcnq1*. Note that ~130 kb of the longest isoform are deleted in *Del*^{7AI}.

(*v*) UCSC genes annotated in the region, including *Ascl2*, located immediately distal of the *Del*^{7AI} breakpoint.

(**B**) RT-PCR detection of *Kcnq1ot1* at 0.3, 202, and 307 kb downstream of the TSS, on E13.5 placental RNA from two +/*Del*^{7AI} and one wild-type control conceptuses. PCRs were performed on total RNA samples, with (+) or without (-) reverse transcriptase (RT) priming of cDNA with random primers (N_{15}). C^-: water control. C^+: genomic DNA. The molecular weight ladder is the exACTGene 100bp ladder (Fisher Scientific).
