# Supplementary material for: Transcription factor ASCL2 is required for development of the glycogen trophoblast cell lineage
Source: PLoS Genet. 2018 Aug 10;14(8):e1007587. doi: 10.1371/journal.pgen.1007587 (PMC6105033; doi:10.1371/journal.pgen.1007587)
Supplement: S3 Fig — (A) Igf2 RT-qPCR on wild type and +/Del7AI E13.5 placental cDNA. Expression is relative to Ppia. Three biological replicates for each genotype were analysed. Graphs show mean ± SD. (B) Igf2 ISH on frozen sections of wild type and +/Del7AI E13.5 placentae. Multiple sections from two placentae of each genotype were assessed and representative pictures are shown. The sense probe gave no signal (not shown). The blue stain shows Igf2 expression, mostly in the junctional zone and GlyT cells in the decidua. Scale bar: 0.5 mm. jz, junctional zone; lab: labyrinth; dec, decidua. (PDF) [file pgen.1007587.s003.pdf]

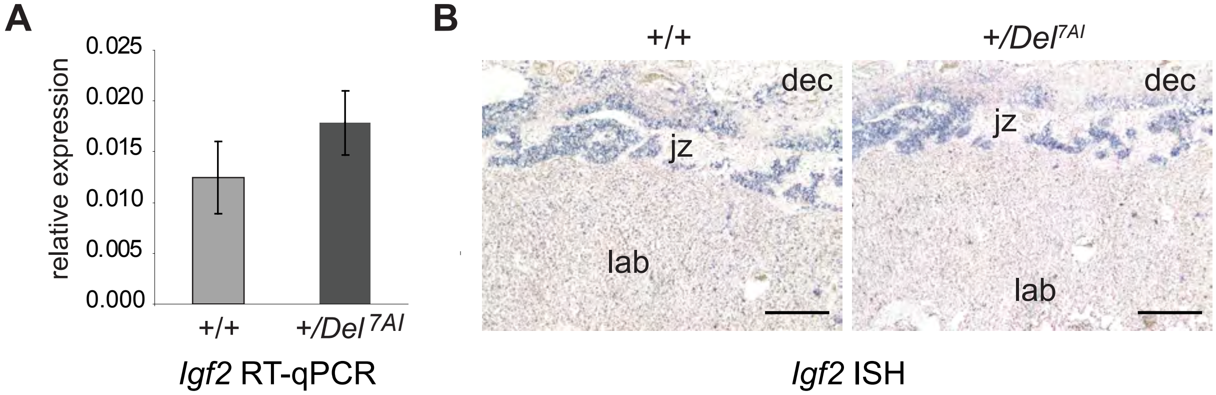

S3 Fig. Paternal *Igf2* expression is unaffected in +/*Del*^{7AI} placentae at E13.5.
(A) *Igf2* RT-qPCR on wild type and +/*Del*^{7AI} E13.5 placental cDNA. Expression is relative to *Ppia*. Three biological replicates for each genotype were analysed. Graphs show mean ± SD.
(B) *Igf2* ISH on frozen sections of wild type and +/*Del*^{7AI} E13.5 placentae. Multiple sections from two placentae of each genotype were assessed and representative pictures are shown. The sense probe gave no signal (not shown). The blue stain shows *Igf2* expression, mostly in the junctional zone and GlyT cells in the decidua. Scale bar: 0.5 mm. jz, junctional zone; lab: labyrinth; dec, decidua.
