# Supplementary material for: Transcription factor ASCL2 is required for development of the glycogen trophoblast cell lineage
Source: PLoS Genet. 2018 Aug 10;14(8):e1007587. doi: 10.1371/journal.pgen.1007587 (PMC6105033; doi:10.1371/journal.pgen.1007587)
Supplement: S4 Fig — (A) Trophoblast stem cell (TSC) lines of the given genotypes were differentiated for 2 days by FGF4 withdrawal and Ascl2 levels, normalized to Ppia levels, were measured by RT-qPCR. In paternal deletion mutants (+/Del7AI), total Ascl2 levels are increased by 1.6-fold over wild-type TSCs (*, p<0.05). Graphs show mean + SD. The numbers of independent TSC lines of each genotype analysed (biological replicates) are given at the bottom (n =). (B) Relative levels of Ascl2 and Phlda2 in E13.5 wild-type and Ascl2lacZ/Del7AI rescued placentae, determined as described in A. Three samples of each genotype were analysed and graphs show mean ± SD of biological triplicates (**, p = 0.0003). (PDF) [file pgen.1007587.s004.pdf]

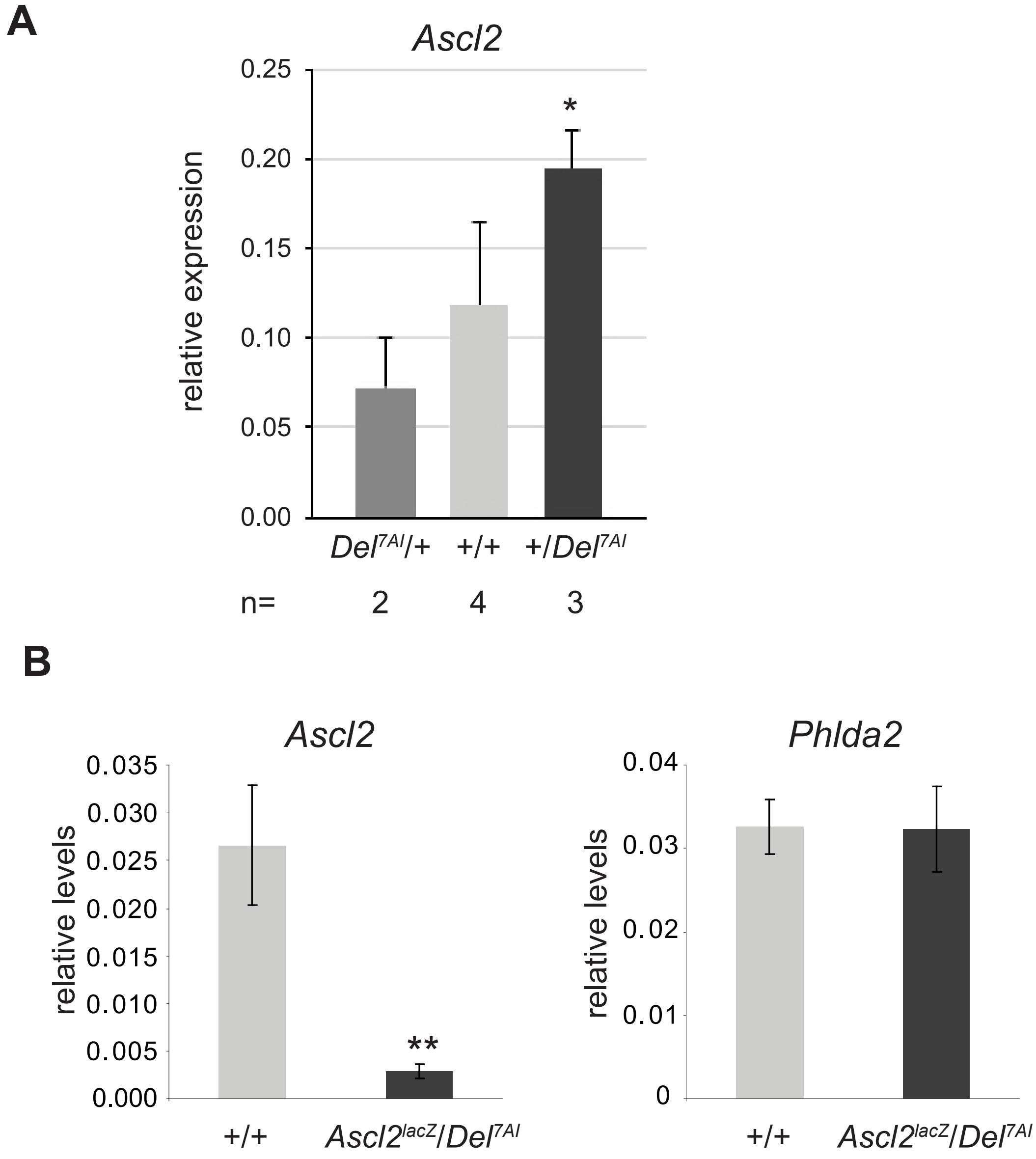

S4 Fig. Effect of *Del*^{7AI} on *Ascl2* mRNA levels in differentiated TSCs and rescued placentae. **(A)** Trophoblast stem cell (TSC) lines of the given genotypes were differentiated for 2 days by FGF4 withdrawal and *Ascl2* levels, normalized to *Ppia* levels, were measured by RT-qPCR. In paternal deletion mutants (+/*Del*^{7AI}), total *Ascl2* levels are increased by 1.6-fold over wild-type TSCs (*, $p<0.05$). Graphs show mean + SD. The numbers of independent TSC lines of each genotype analysed (biological replicates) are given at the bottom (n=). **(B)** Relative levels of *Ascl2* and *Phlda2* in E13.5 wild-type and *Ascl2*^{lacZ}/*Del*^{7AI} rescued placentae, analysed as described in **A**. Three samples of each genotype were analysed and graphs show mean ± SD of biological triplicates (**, $p=0.0003$).
