# Supplementary material for: Transcription factor ASCL2 is required for development of the glycogen trophoblast cell lineage
Source: PLoS Genet. 2018 Aug 10;14(8):e1007587. doi: 10.1371/journal.pgen.1007587 (PMC6105033; doi:10.1371/journal.pgen.1007587)
Supplement: S5 Fig — Frozen sections of E15.5 placentae of the given genotypes were analysed for the expression of Prl3b1 and Gcm1 by ISH. The basement membrane marker laminin was detected by IHC on paraffin sections. Scale bar: 0.5 mm. Spt, spongiotrophoblast cells; dec, decidua; P-TGC, parietal trophoblast giant cells; lab, labyrinthine layer. (PDF) [file pgen.1007587.s005.pdf]

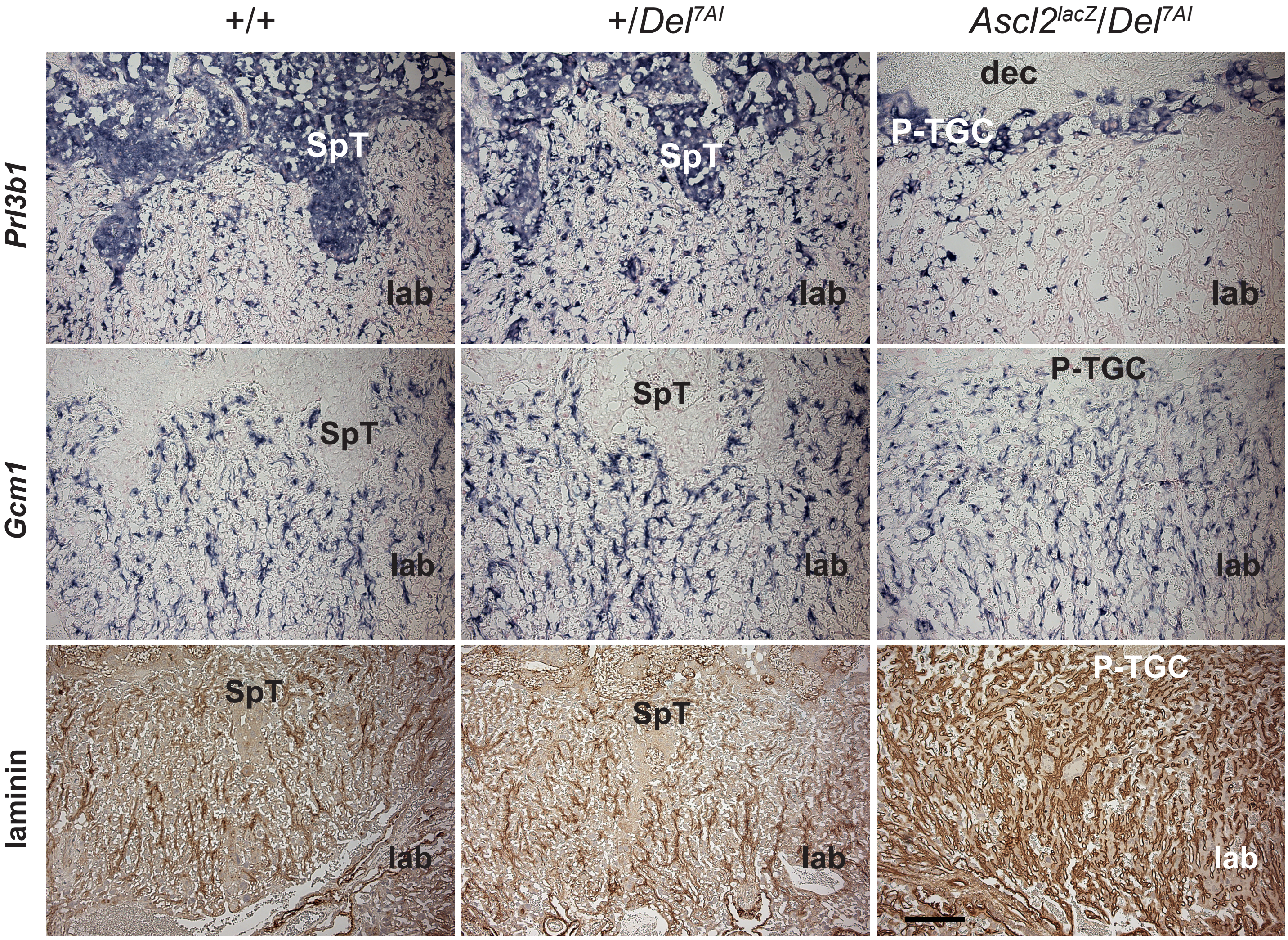

S5 Fig. Abnormal labyrinth development in *Ascl2^{lacZ}/Del7AI* placentae at E15.5.
Frozen sections of E15.5 placentae of the given genotypes were analysed for the expression of *Prl3b1* and *Gcm1* by ISH. The basement membrane marker laminin was analysed by IHC on paraffin sections. Scale bar: 0.5 mm. Spt, spongiotrophoblast cells; dec, decidua; P-TGC, parietal trophoblast giant cells; lab, labyrinthine layer.
