# Supplementary material for: Transcription factor ASCL2 is required for development of the glycogen trophoblast cell lineage
Source: PLoS Genet. 2018 Aug 10;14(8):e1007587. doi: 10.1371/journal.pgen.1007587 (PMC6105033; doi:10.1371/journal.pgen.1007587)
Supplement: S6 Fig — Adjacent sections of the Ascl2lacZ/+ E8.5 conceptuses analysed in Fig 6B were treated as described in this figure but without incubation with the anti-PCDH12 primary antibodies. Punctate staining for the secondary antibody (arrow) is still visible above the giant cell layer, within the decidua. P-TGC, parietal trophoblast giant cells; dec, decidua; ch, chorion. (PDF) [file pgen.1007587.s006.pdf]

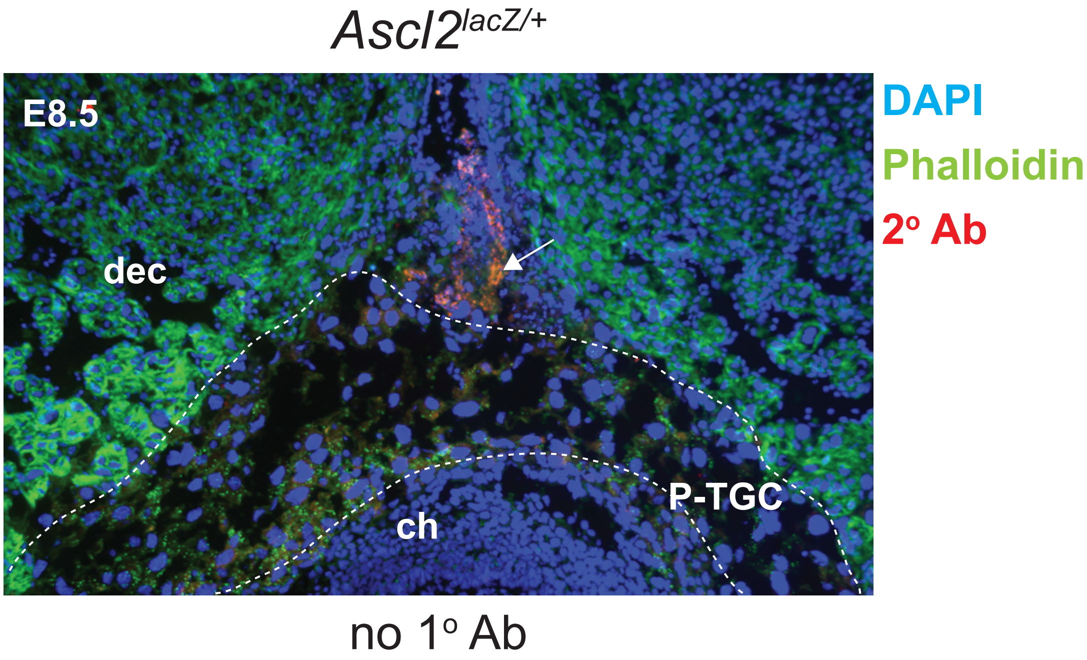

S6 Fig. Primary antibody-independent staining in the decidua.
Adjacent sections of the *Ascl2*$^{lacZ/+}$ E8.5 conceptuses analysed in Fig. 6B were treated as described in this figure but without incubation with the anti-PCDH12 primary antibodies. Punctate staining for the secondary antibody (arrow) is still visible above the giant cell layer, within the decidua. P-TGC, parietal trophoblast giant cells; dec, decidua; ch, chorion.
