# Supplementary material for: Transcription factor ASCL2 is required for development of the glycogen trophoblast cell lineage
Source: PLoS Genet. 2018 Aug 10;14(8):e1007587. doi: 10.1371/journal.pgen.1007587 (PMC6105033; doi:10.1371/journal.pgen.1007587)
Supplement: S7 Fig — (A) Cell-cycle distribution of wild-type and Ascl2lacZ/+ mutant TSCs as monitored by flow cytometry using propidium iodide staining. Profiles were generated on the indicated days following FGF4 and conditioned medium withdrawal. 2n marks diploid cells in G1 phase, whereas 4n represents a mixture of G2-phase diploid and G1-phase tetraploid cells. Endoreduplication is clearly seen at higher ploidies. (B) Images of wild-type and Ascl2lacZ/+ mutant TSCs at d0 and d4 of differentiation. Scale bar, 100 μm. Refers to data presented in Fig 8B. (PDF) [file pgen.1007587.s007.pdf]

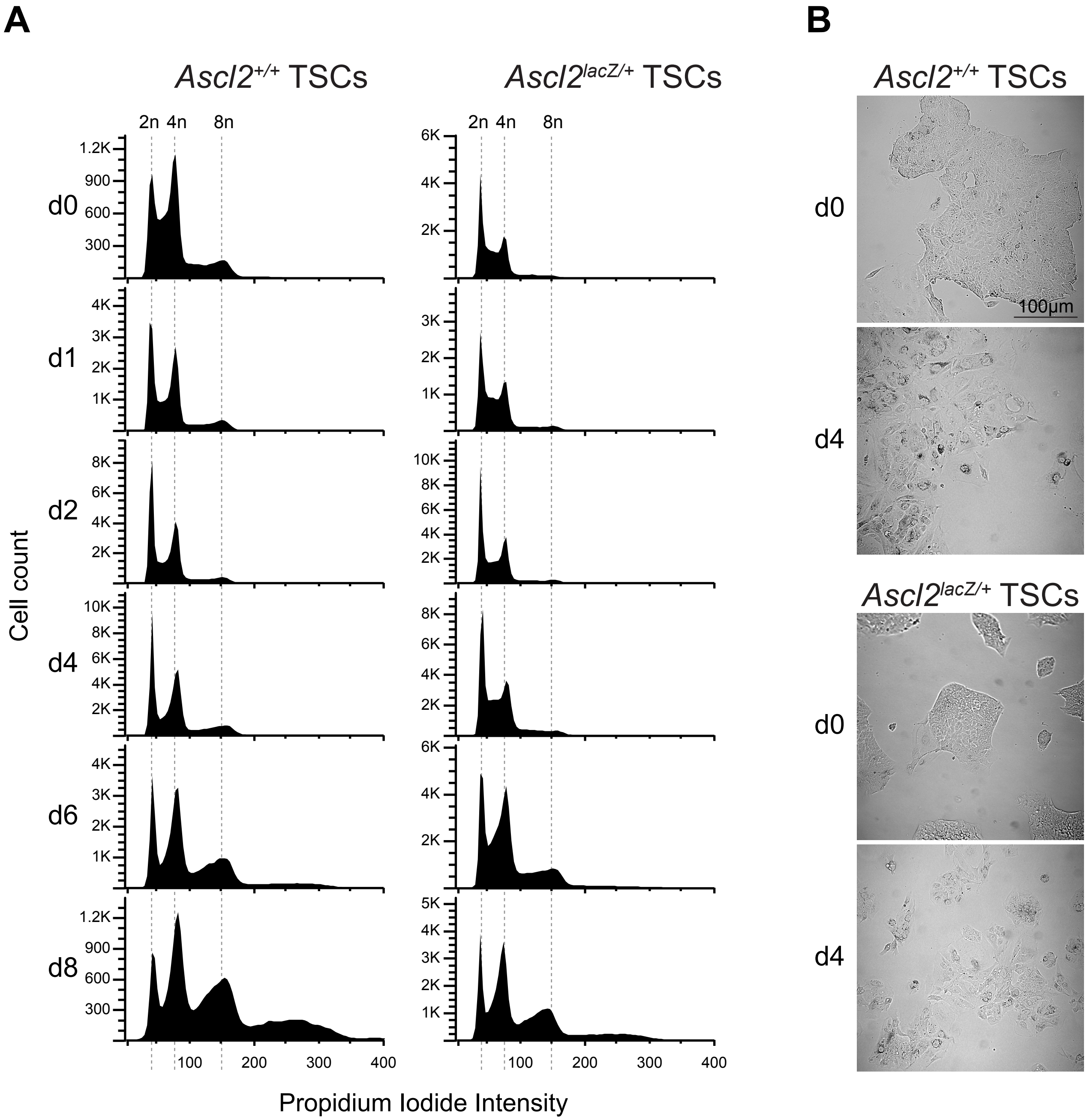

S7 Fig. Endoreduplication of differentiating wild-type and *Ascl2*$^{lacZ/+}$ TSCs.
(**A**) Cell-cycle distribution of wild-type and *Ascl2*$^{lacZ/+}$ mutant TSCs as monitored by flow cytometry using propidium iodide staining. Profiles were generated on the indicated days following FGF4 and conditioned medium withdrawal. 2n marks diploid cells in G1 phase, whereas 4n represents a mixture of G2-phase diploid and G1-phase tetraploid cells. Endoreduplication is clearly seen at higher ploidies. (**B**) Images of wild-type and *Ascl2*$^{lacZ/+}$ mutant TSCs at d0 and d4 of differentiation. Scale bar, 100 µm. Refers to data presented in Fig. 8B.
