# Supplementary material for: Transcription factor ASCL2 is required for development of the glycogen trophoblast cell lineage
Source: PLoS Genet. 2018 Aug 10;14(8):e1007587. doi: 10.1371/journal.pgen.1007587 (PMC6105033; doi:10.1371/journal.pgen.1007587)
Supplement: S8 Fig — For each genotype, the approximate total Ascl2 mRNA levels are presented as a percentage of the wild-type levels, set at 100%. (PDF) [file pgen.1007587.s008.pdf]

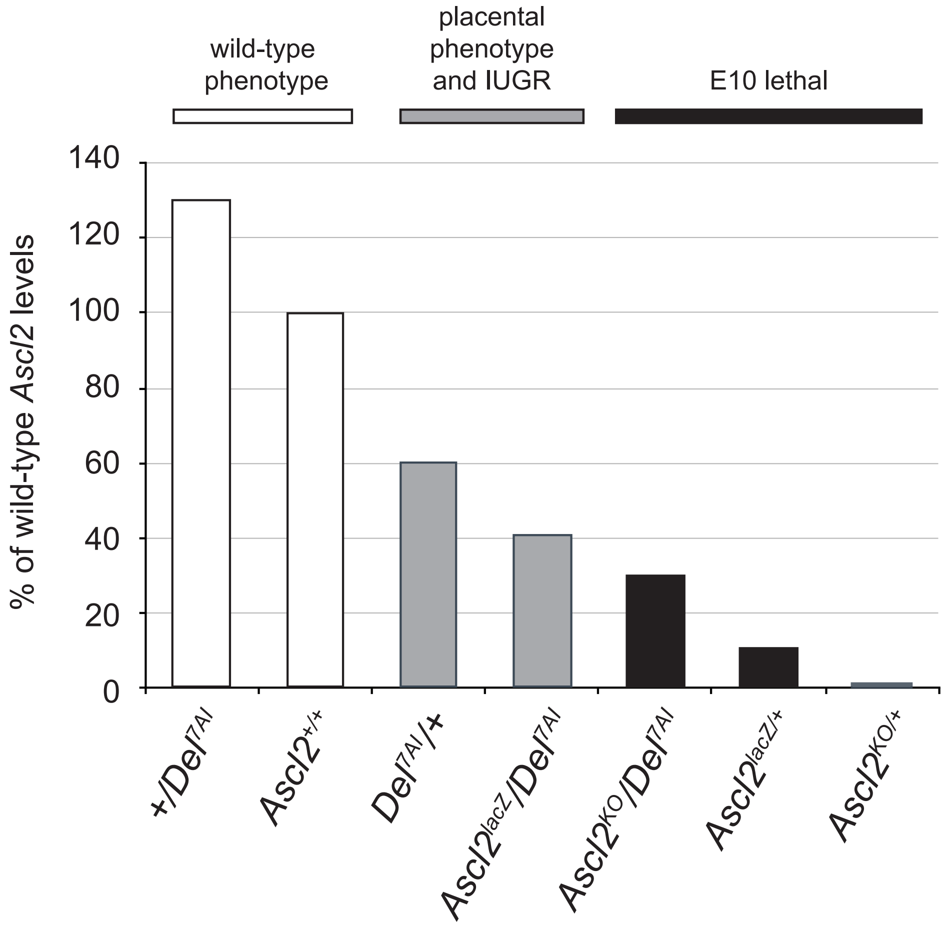

S8 Fig. Dosage-sensitive effects of *Ascl2* mRNA levels on placental phenotype.
For each genotype, the approximate total *Ascl2* mRNA levels are presented as a percentage of the wild-type levels, set at 100%.
