# Supplementary material for: Transcription factor ASCL2 is required for development of the glycogen trophoblast cell lineage
Source: PLoS Genet. 2018 Aug 10;14(8):e1007587. doi: 10.1371/journal.pgen.1007587 (PMC6105033; doi:10.1371/journal.pgen.1007587)
Supplement: S9 Fig — (A) Structure of the IC1-IC2 imprinted domains on distal mouse Chr7, drawn to scale. Paternally expressed genes are in blue, maternally expressed genes in red. Two isoforms of Kcnq1ot1 have been described; the more stable form terminates within intron 10 of Kcnq1, whereas a longer form has been detected, extending all the way past Th, a gene maternally expressed in placenta from the LTR RMER19A (Jones, 2011). (B) The Tel7KI allele carrying a pCAGGS-EGFP reporter inserted upstream of Ins2. The EGFP is imprinted and maternally expressed in the embryo in a Kcnq1ot1-dependent manner (Jones, 2011). (C) Del7AI allele, showing partial LOI at Ascl2 and Tssc4 upon paternal transmission. (D) YAC transgene showing appropriate imprinting of the IC2 domain, except at Ascl2 and Tssc4 (Cerrato, 2005). In both C and D, the 3’ end structure of the longer Kcnq1ot1 isoform is unknown (question marks). (PDF) [file pgen.1007587.s009.pdf]

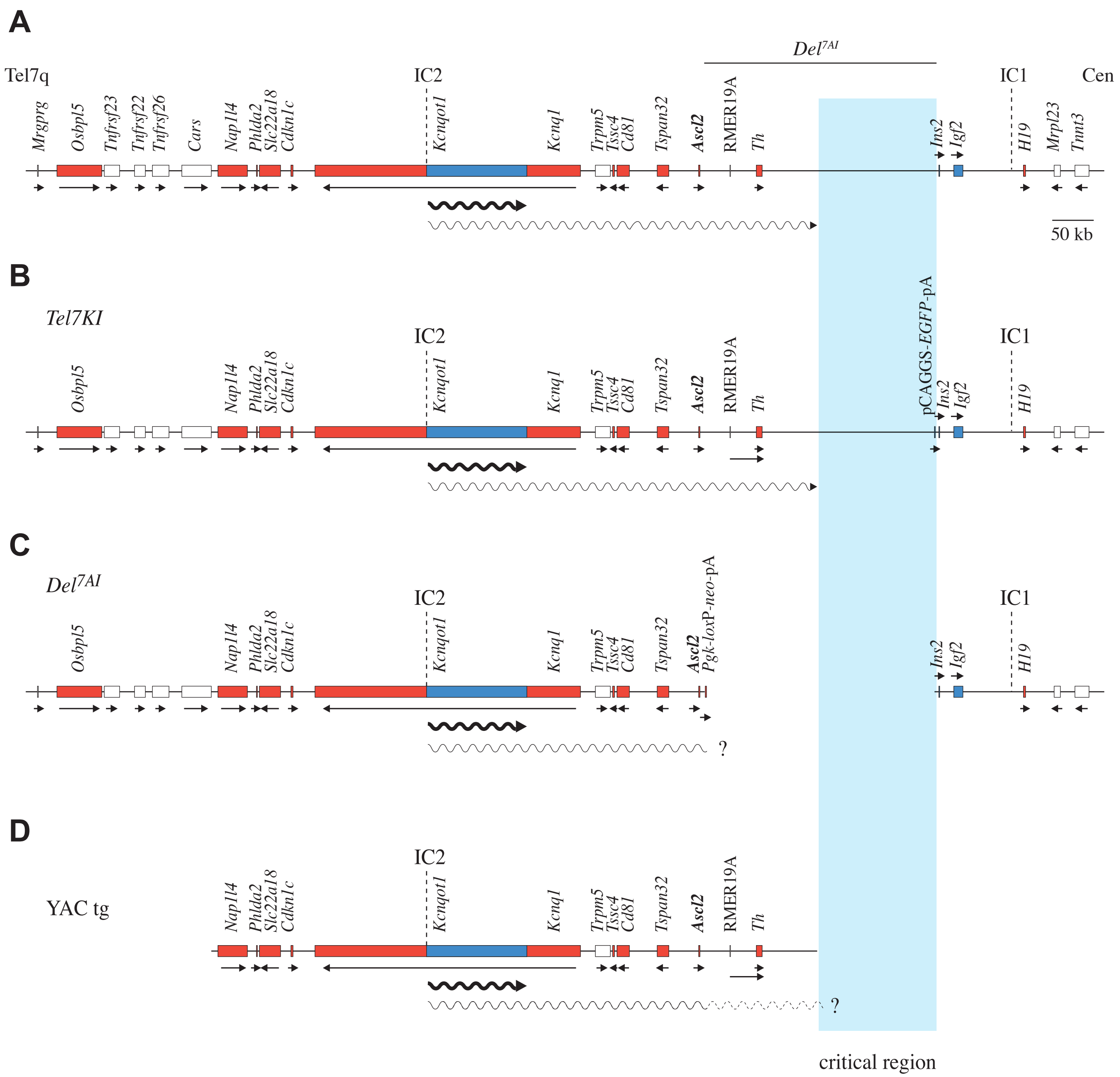

S9 Fig. Potential critical region for extended *Kcnq1ot1* silencing.
(**A**) Structure of the IC1-IC2 imprinted domains on distal mouse Chr7, drawn to scale. Paternally-expressed genes are in blue, maternally-expressed genes in red, and biallelically-expressed genes in white. Two main isoforms of *Kcnq1ot1* have been described: the more stable form (thick wavy line) terminates within intron 10 of *Kcnq1*, whereas a longer form has been detected, extending all the way past *Th*, a gene maternally expressed in placenta from the LTR RMER19A (Jones, 2011).
(**B**) The Tel7KI allele carrying a pCAGGS-EGFP reporter inserted upstream of *Ins2*. The EGFP is imprinted and maternally expressed in the embryo in a *Kcnq1ot1*-dependent manner (Jones, 2011).
(**C**) *Del*^{7AI} allele, showing partial LOI at *Ascl2* and *Tssc4* upon paternal transmission.
(**D**) YAC transgene showing appropriate imprinting of the IC2 domain, except at *Ascl2* and *Tssc4* (Cerrato, 2005).
In both **C** and **D**, the 3' end structure of the longer *Kcnq1ot1* isoform is unknown (question marks).
