# Supplementary material for: Transcription factor ASCL2 is required for development of the glycogen trophoblast cell lineage
Source: PLoS Genet. 2018 Aug 10;14(8):e1007587. doi: 10.1371/journal.pgen.1007587 (PMC6105033; doi:10.1371/journal.pgen.1007587)
Supplement: S4 Table — (PDF) [file pgen.1007587.s013.pdf]

S4 Table. Primers used in this study.

Gene	Purpose	Name	Sequence (5' to 3')	Reference
Ascl2	allele-specific	1148F	GGCTGTTAACACCCGCTACTCCG	this study
		129R	TCTTGCCCTCAGGGCCCAACCG	this study
		726R	ACAGCAGGGTTCCCACACTGG	1
	genomic	in2F1	CGGTGTCCGTACACATTGACAC	this study
Igf2	ISH probe	Igf2 F	CCTCGTCACTTCTCCTACG	2
		Igf2 R	CGTTTGGCCTCTCTGAACTC	2
Cdkn1c	allele-specific	p57S	GCCAATGCGAACGACTTC	3
		p574	TACACCTTGGGACCAGCGTACTCC	3
Tssc4	allele-specific	F1	AACCAGTGTGAGTGACAATGGC	4
		R35	AAAGGCCTTCGAGGTCCCCTG	4
Phlda2	allele-specific	Ipl 1	GGTGCAACCTTTCGAAATGG	this study
		Ipl R2	CAGCAAGCACGGGAATATCT	this study
Ascl2	RT-qPCR	Ascl2 qF1	TCCTGGTGGACCTACCTGCTT	5
		Ascl2 qR1	AGGTCAGTCAGCACTTGGCATT	5
Cdkn1c	RT-qPCR	Cdkn1c qF	GCGCAAACGTCTGAGATGAG	5
		Cdkn1c qR	CAGCCGAAGCCCAGAGTTC	5
Tssc4	RT-qPCR	Tssc4 qF2	ACGGGTGTCAGGTCGTATGG	5
		Tssc4 qR2	TGAGGGAGACGGTGTCAGAAG	5
Phlda2	RT-qPCR	Phlda2 qF	CCCGCCAAGGAGCTGTTT	5
		Phlda2 qR	CCTTGTAATAGTTGGTGACGATGGT	5
Ppia	RT-qPCR	Ppia F	CGCGTCTCCTTCGAGCTGTTTG	6
		Ppia R	TGTAAAGTCACCACCCTGGCACAT	6
Pcdh12	RT-qPCR	Pcdh12 qF4	CCAGCACTGGCTTTGATCTG	this study
		Pcdh12 qR4	CGACAGTTGTAGGCCCTATTATCC	this study
Tpbpa	RT-qPCR	Tpbpa qF1	CAGCTTTGGACATCACAGGTACTT	5
		Tpbpa qR1	TGCGCTTCAGGGACTATAGCA	5
Cdx2	RT-qPCR	Cdx2 qF1	TCCCTCGTCTTTGGCTGAAG	this study
		Cdx2 qR1	GGCTTGTTTGGCTCGTTACAC	this study
Igf2	RT-qPCR	Igf2 qPCR F	CAGCGGCAGCACAGATTTT	7
		Igf2 qPCR R	TCCTGGACACGGGAGCAA	7
Kcnq1ot1	RT-PCR	0.3kF/F239	GGTTTTTCACGGTGAGGTCATATCA	M.J. Higgins
		0.3kR/R240	GGAGGTCTAGGCTCAGGACAAACACT	M.J. Higgins
		202kF	GCCAAGAGGGTACTAAGGTC	8
		202kR	ACGTCTAGCATCCATGAGG	8
		307kF	ACACAGAGGTTTCCCCATCA	8
		307kR	GGAGTCAGTGTAGTGCCTATGG	8

References

1. **Oh, R., Ho, R., Mar, L., Gertsenstein, M., Paderova, J., Hsien, J., Squire, J.A., Higgins, M., Nagy, A., Lefebvre, L.** (2008). Epigenetic and phenotypic consequences of a truncation disrupting the imprinted domain on distal mouse chromosome 7. *Mol. Cell. Biol.* **28**, 1092-1103.

2. **GenePaint Database** [http://www.GenePaint.org], RNA probe 486.

3. **Jones, M.J., Lefebvre, L.** (2009). An imprinted GFP insertion reveals long-range epigenetic regulation in embryonic lineages. *Dev. Biol.* **336**, 42-52.

4. **Paulsen, M., El-Maarri, O., Engemann, S., Strödicke, M., Franck, O., Davies, K., Reinhardt, R., Reik, W., Walter, J.** (2000). Sequence conservation and variability of imprinting in the Beckwith-Wiedemann syndrome gene cluster in human and mouse. *Hum. Mol. Genet.* **9**, 1829-1841.

5. **Oh-McGinnis, R., Bogutz, A.B., Lee, K.Y., Higgins, M., Lefebvre, L.** (2010). Rescue of placental phenotype in a mechanistic model of Beckwith-Wiedemann syndrome. *BMC Dev. Biol.* **10**, 50.

6. **Mamo, S., Gal, A.B., Bodo, S., Dinnyes, A.** (2007). Quantitative evaluation and selection of reference genes in mouse oocytes and embryos cultured in vivo and in vitro. *BMC Dev. Biol.* **7**, 14.

7. **Oh-McGinnis, R., Bogutz, A.B., Lefebvre, L.** (2011). Partial loss of *Ascl2* function affects all three layers of the mature placenta and causes intrauterine growth restriction. *Dev. Biol.* **351**, 277-286.

8. **Golding, M.C., Magri, L.S., Zhang, L., Lalone, S.A., Higgins, M.J., Mann, M.R.W.** (2011) Depletion of *Kcnq1ot1* non-coding RNA does not affect imprinting maintenance in stem cells. *Development* **138**, 3667–3678.
